# Supplementary material for: Concerted catalysis of single atom and nanocluster enhances bio-ethanol activation and dehydrogenation
Source: Nat Commun. 2025 Apr 26;16:3935. doi: 10.1038/s41467-025-59127-0 (PMC12033288; doi:10.1038/s41467-025-59127-0)
Supplement: Supplementary file 1 — Supplementary Information [file 41467_2025_59127_MOESM1_ESM.pdf]

Concerted catalysis of single atom and nanocluster enhances bio-ethanol activation and dehydrogenation

Zhao Sun [1, 2], Weizhi Shi [1], Louise R. Smith [2], Nicholas F. Dummer [2], Haifeng Qi [2], Zhiqiang Sun [1*], Graham J. Hutchings [2*]

[1]Hunan Engineering Research Center of Clean and Low-Carbon Energy Technology, School of Energy Science and Engineering, Central South University, Changsha 410083, China

[2]Max Planck–Cardiff Centre on the Fundamentals of Heterogeneous Catalysis FUNCAT, Cardiff Catalysis Institute, School of Chemistry, Cardiff University, Cardiff CF24 4HQ, United Kingdom

✉email:

zqsun@csu.edu.cn (Zhiqiang Sun)

Hutch@cardiff.ac.uk (Graham J. Hutchings)

The Supporting Information includes:

Supplementary Figure 1. Textural measurements of as-prepared Ni-based catalysts. a, b N_2 adsorption-desorption and pore size distribution of Ni/MTAC catalysts. c, d N_2 adsorption-desorption and pore size distribution of 1Ni/MTC, 1Ni/Al_2O_3, 1Ni/SiO_2, and 1Ni/CeO_2 catalysts.

Supplementary Figure 2. SEM images of as-prepared Ni/MTAC catalysts. a, 0.5Ni/MTAC. b 0.75Ni/MTAC. c 1.25Ni/MTAC. d 1.5Ni/MTAC.

Supplementary Figure 3. a-b TEM. c-d high-resolution-TEM. e EELS analysis of 1Ni/MTAC on nanoclusters in a bright area of 1Ni/MTAC.

Supplementary Figure 4. Active site distributions of 0.5, 1, 1.5Ni/MTAC.

Supplementary Figure 5. Size distribution of 1Ni/MTAC catalyst.

Supplementary Figure 6. EDS mapping results of 1Ni/MTAC sample for Ni, Mo, Ti, and Al elements.

Supplementary Figure 7. The Ni 2*p* XPS spectra of the as-prepared 1Ni/support catalysts. a 1Ni/MTC. b 1Ni/Al_2O_3. c 1Ni/SiO_2. d 1Ni/CeO_2.

Supplementary Figure 8. Raman spectra of the as-prepared Ni/MTAC catalysts.

Supplementary Figure 9. The XPS spectra of the as-prepared 0.5Ni/MTAC catalyst. a Ti 2*p* spectrum. b C 1*s* spectrum. c Al 2*p* spectrum. d O 1*s* spectrum.

Supplementary Figure 10. XPS spectra of the as-prepared 0.75Ni/MTAC catalyst. a Ti 2*p* spectrum. b C 1*s* spectrum. c Al 2*p* spectrum. d O 1*s* spectrum.

Supplementary Figure 11. XPS spectra of the as-prepared 1Ni/MTAC catalyst. a Ti 2*p* spectrum. b C 1*s* spectrum. c Al 2*p* spectrum. d O 1*s* spectrum.

Supplementary Figure 12. XPS spectra of the as-prepared 1.25Ni/MTAC catalyst. a Ti 2*p* spectrum. b C 1*s* spectrum. c Al 2*p* spectrum. d O 1*s* spectrum.

Supplementary Figure 13. XPS spectra of the as-prepared 1.5Ni/MTAC catalyst. a Ti 2*p* spectrum. b C 1*s* spectrum. c Al 2*p* spectrum. d O 1*s* spectrum.

Supplementary Figure 14. XAFS analysis of as-prepared samples. a Fourier transform of Ni K-edge EXAFS spectra in K-space. The fitting results of Ni K-edge Fourier-transform EXAFS spectra on K-space of b 0.5Ni/MTAC, c 1Ni/MTAC, and d 1.5Ni/MTAC.

Supplementary Figure 15. The fitting results of Ni K-edge Fourier-transform EXAFS spectra on R-space of a 0.5Ni/MTAC, b 1Ni/MTAC, and c 1.5Ni/MTAC.

Supplementary Figure 16. XAFS analysis of as-prepared samples. a Fourier transform of Mo K-edge EXAFS spectra in K-space. The fitting results of Mo K-edge Fourier-transform EXAFS spectra on K-space of b 0.5Ni/MTAC, c 1Ni/MTAC, and d 1.5Ni/MTAC.

Supplementary Figure 17. The fitting results of Mo K-edge Fourier-transform EXAFS spectra on R-space of a 0.5Ni/MTAC, b 1Ni/MTAC, and c 1.5Ni/MTAC.

Supplementary Figure 18. Wavelet transformed EXAFS spectra. Ni K-edge EXAFS spectra for a Ni foil. b 0.5Ni/MTAC. c 1Ni/MTAC, and d 1.5Ni/MTAC.

Supplementary Figure 19. Wavelet transformed EXAFS spectra. Mo K-edge EXAFS spectra for a Mo_2C. b 0.5Ni/MTAC. c 1Ni/MTAC, and d 1.5Ni/MTAC.

Supplementary Figure 20. SRE performance of the 0.5Ni/MTAC. Activity test conditions: 1 atm, 550 °C, S/E=3, liquid feeding rate 0.005 mL min^{-1}, N_2 flow rate 40 mL min^{-1}.

Supplementary Figure 21. SRE performance of the 0.75Ni/MTAC. Activity test conditions: 1 atm, 550 °C, S/E=3, liquid feeding rate 0.005 mL min^{-1}, N_2 flow rate 40 mL min^{-1}.

Supplementary Figure 22. SRE performance of the 1.25Ni/MTAC. Activity test conditions: 1 atm, 550 °C, S/E=3, liquid feeding rate 0.005 mL min^{-1}, N_2 flow rate 40 mL min^{-1}.

Supplementary Figure 23. SRE performance of the 1.5Ni/MTAC. Activity test conditions: 1 atm, 550 °C, S/E=3, liquid feeding rate 0.005 mL min^{-1}, N_2 flow rate 40 mL min^{-1}.

Supplementary Figure 24. MS signals from the operando pulse experiment of ethanol and water (S/E=3) over 1Ni/MTAC catalyst at 550 °C.

Supplementary Figure 25. Estimated total active site numbers of 0.5Ni/MTAC, 1Ni/MTAC, and 1.5Ni/MTAC catalysts.

Supplementary Figure 26. SRE performance of 1Ni/MTAC at 450 °C. Activity test conditions: 1 atm, S/E=3, liquid feeding rate 0.005 mL min^{-1}, N_2 flow rate 40 mL min^{-1}.

Supplementary Figure 27. SRE performance of 1Ni/MTAC at 500 °C. Activity test conditions: 1 atm, S/E=3, liquid feeding rate 0.005 mL min^{-1}, N_2 flow rate 40 mL min^{-1}.

Supplementary Figure 28. SRE performance of 1Ni/MTAC at 600 °C. Activity test conditions: 1 atm, S/E=3, liquid feeding rate 0.005 mL min^{-1}, N_2 flow rate 40 mL min^{-1}.

Supplementary Figure 29. SRE performance of 1Ni/MTAC at GHSV values for 2890 h^{-1}. Activity test conditions: 1 atm, 550 °C, S/E=3, liquid feeding rate 0.005 mL min^{-1}, N_2 flow rate 30 mL min^{-1}.

Supplementary Figure 30. SRE performance of 1Ni/MTAC at GHSV values for 3316 h^{-1}. Activity test conditions: 1 atm, 550 °C, S/E=3, liquid feeding rate 0.005 mL min^{-1}, N_2 flow rate 35 mL min^{-1}.

Supplementary Figure 31. SRE performance of 1Ni/MTAC at GHSV values for 4168 h^{-1}. Activity test conditions: 1 atm, 550 °C, S/E=3, liquid feeding rate 0.005 mL min^{-1}, N_2 flow rate 45 mL min^{-1}.

Supplementary Figure 32. SRE performance of 1Ni/MTAC at different liquid feeding rates. (The GHSV values for 3742 h^{-1}, 4076 h^{-1}, 6745 h^{-1}, and 36775 h^{-1} are 0.005, 0.01, 0.05, and 0.5 liquid feeding rate mL·min^{-1}) Activity test conditions: 1 atm, 550 °C, S/E=3, N_2 flow rate 40 mL min^{-1}.

Supplementary Figure 33. Unit reaction rate of 1Ni/MTAC and 10Ni/MTAC. Activity test conditions: 1 atm, 550 °C, S/E=3, liquid feeding rate 0.005 mL min^{-1}, N_2 flow rate 35 mL min^{-1}.

Supplementary Figure 34. Morphology characterization of the 120 h reacted 1Ni/MTAC catalyst. a SEM image, b TEM image.

Supplementary Figure 35. SRE performance of 1Ni/MTC. Activity test conditions: 1 atm, 550 °C, S/E=3, liquid feeding rate 0.005 mL min^{-1}, N_2 flow rate 40 mL min^{-1}.

Supplementary Figure 36. SRE performance of 1Ni/Al_2O_3. Activity test conditions: 1

atm, 550 °C, S/E=3, liquid feeding rate 0.005 mL min^{-1}, N_2 flow rate 40 mL min^{-1}.

Supplementary Figure 37. SRE performance 1Ni/SiO_2. Activity test conditions: 1 atm, 550 °C, S/E=3, liquid feeding rate 0.005 mL min^{-1}, N_2 flow rate 40 mL min^{-1}.

Supplementary Figure 38. SRE performance of 1Ni/CeO_2. Activity test conditions: 1 atm, 550 °C, S/E=3, liquid feeding rate 0.005 mL min^{-1}, N_2 flow rate 40 mL min^{-1}.

Supplementary Figure 39. XPS spectra of the 120 h reacted 1Ni/MTAC catalyst. a Ni 2*p* spectrum. b Mo 3*d* spectrum. c C 1*s* spectrum. d Ti 2*p* spectrum. e Al 2*p* spectrum. f O 1*s* spectrum.

Supplementary Figure 40. H_2-TPR profiles of unreduced Ni/MTAC catalysts.

Supplementary Figure 41. Comparisons of TPER experimental results at 350 ℃.

Supplementary Figure 42. In situ DRIFTS of 1Ni/MTC.

Supplementary Figure 43. In situ DRIFTS of 1Ni/Al_2O_3.

Supplementary Figure 44. In situ DRIFTS of 1Ni/SiO_2.

Supplementary Figure 45. In situ DRIFTS of 1Ni/CeO_2.

Supplementary Figure 46. DFT studies for CH_3CH_2OH dehydrogenation on Ni_{SA}/MTAC(004). Calculated potential energy diagram and corresponding geometric structures for successive dehydrogenation of ethanol molecule on Ni_{SA}/MTAC(004) . TS represents the transition state. The black and orange numbers denote adsorption energy and reaction energy barrier, respectively.

Supplementary Figure 47. DFT studies for CH_3CH_2OH dehydrogenation on Ni_{NC}/MTAC(004). Calculated potential energy diagram and corresponding geometric structures for successive dehydrogenation of ethanol molecule on Ni_{NC}/MTAC(004) . TS represents the transition state. The black and orange numbers denote adsorption energy and reaction energy barrier, respectively.

Supplementary Figure 48. DFT studies for CH_3CH_2OH dehydrogenation on Ni_{SA-SA}/MTAC(004). Calculated potential energy diagram and corresponding geometric structures for successive dehydrogenation of ethanol molecule on Ni_{SA-SA}/MTAC(004). TS represents the transition state. The black and blue numbers denote adsorption energy

and reaction energy barrier, respectively.

Supplementary Figure 49. DFT studies for CH_3CH_2OH dehydrogenation on Ni_{NC-NC}/MTAC(004). Calculated potential energy diagram and corresponding geometric structures for successive dehydrogenation of ethanol molecule on Ni_{SA-SA}/MTAC(004). TS represents the transition state. The black and blue numbers denote adsorption energy and reaction energy barrier, respectively.

Supplementary Table 1. Physiochemical properties of the as-prepared samples.

Supplementary Table 2. Nominal and actual loading wt% of as-prepared samples.

Supplementary Table 3. Relative proportion of the Ni species for the as-prepared catalysts.

Supplementary Table 4. Ni K-edge XAFS fitted parameters for as-prepared catalysts.

Supplementary Table 5. Mo K-edge XAFS fitted parameters for as-prepared catalysts.

Supplementary Table 6. Catalytic performance for SRE reaction over various Ni based catalysts.

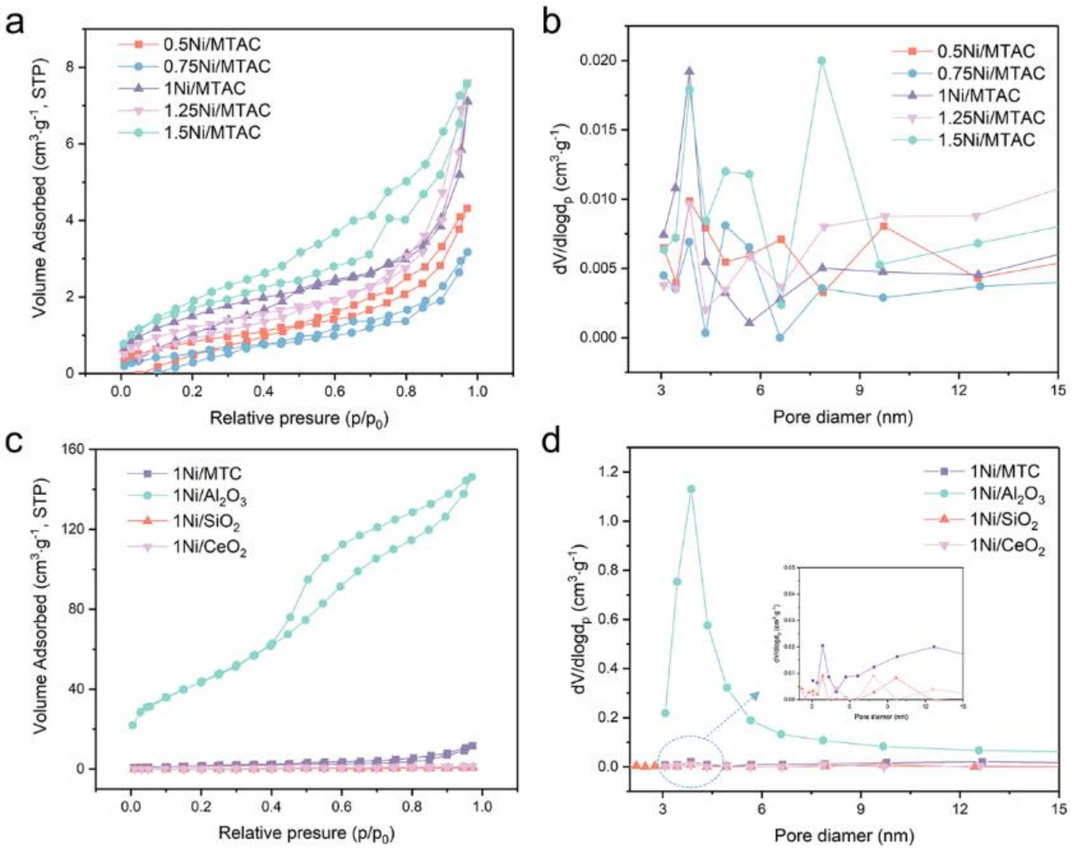

Supplementary Figure 1. Textural measurements of as-prepared Ni-based catalysts. a, **b** N_2 adsorption-desorption and pore size distribution of Ni/MTAC catalysts. **c**, **d** N_2 adsorption-desorption and pore size distribution of 1Ni/MTC, 1Ni/Al_2O_3, 1Ni/SiO_2, and 1Ni/CeO_2 catalysts.

The 1Ni/Al_2O_3 sample displays a type IV isotherm possessing a H1 hysteresis loop, indicating the presence of a characteristic mesoporous structure. All the 1Ni/MTAC samples show a type III isotherm[1]. The increase in the Ni loadings improved the BET surface area of all the Ni/MTAC catalysts (Table S1). Pore size distribution was calculated from the desorption branches of the N_2 isotherms using the BJH method. All the catalysts possess the similar pore size.

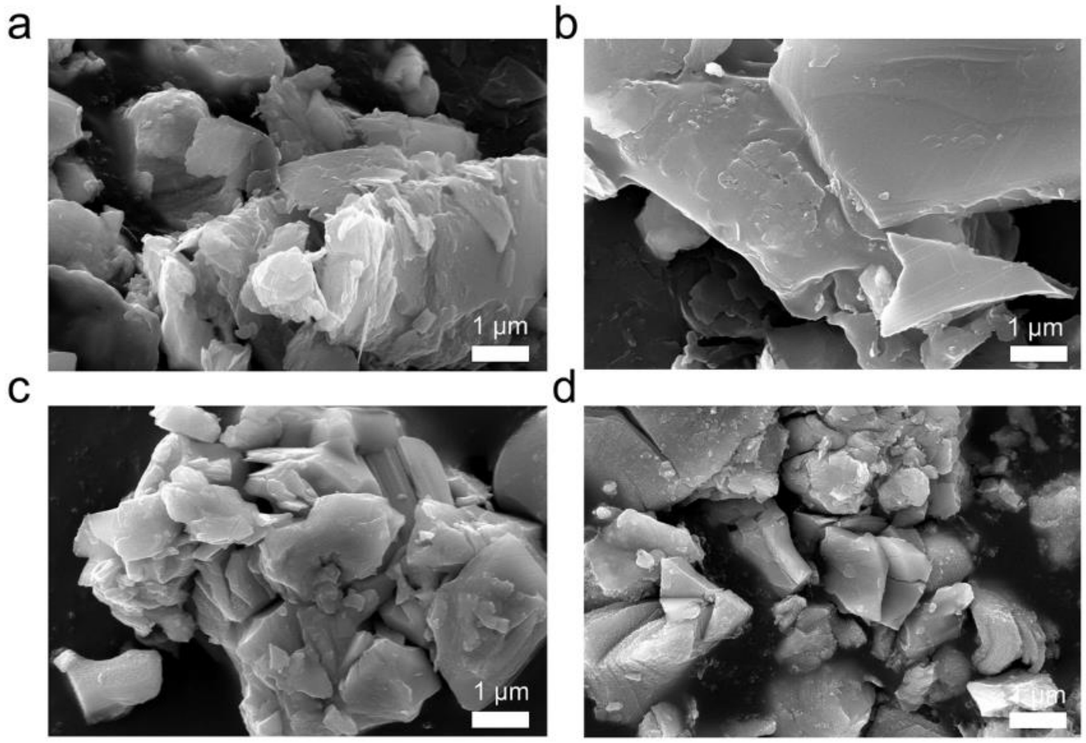

Supplementary Figure 2. SEM images of as-prepared Ni/MTAC catalysts. a 0.5Ni/MTAC. **b** 0.75Ni/MTAC. **c** 1.25Ni/MTAC. **d** 1.5Ni/MTAC.

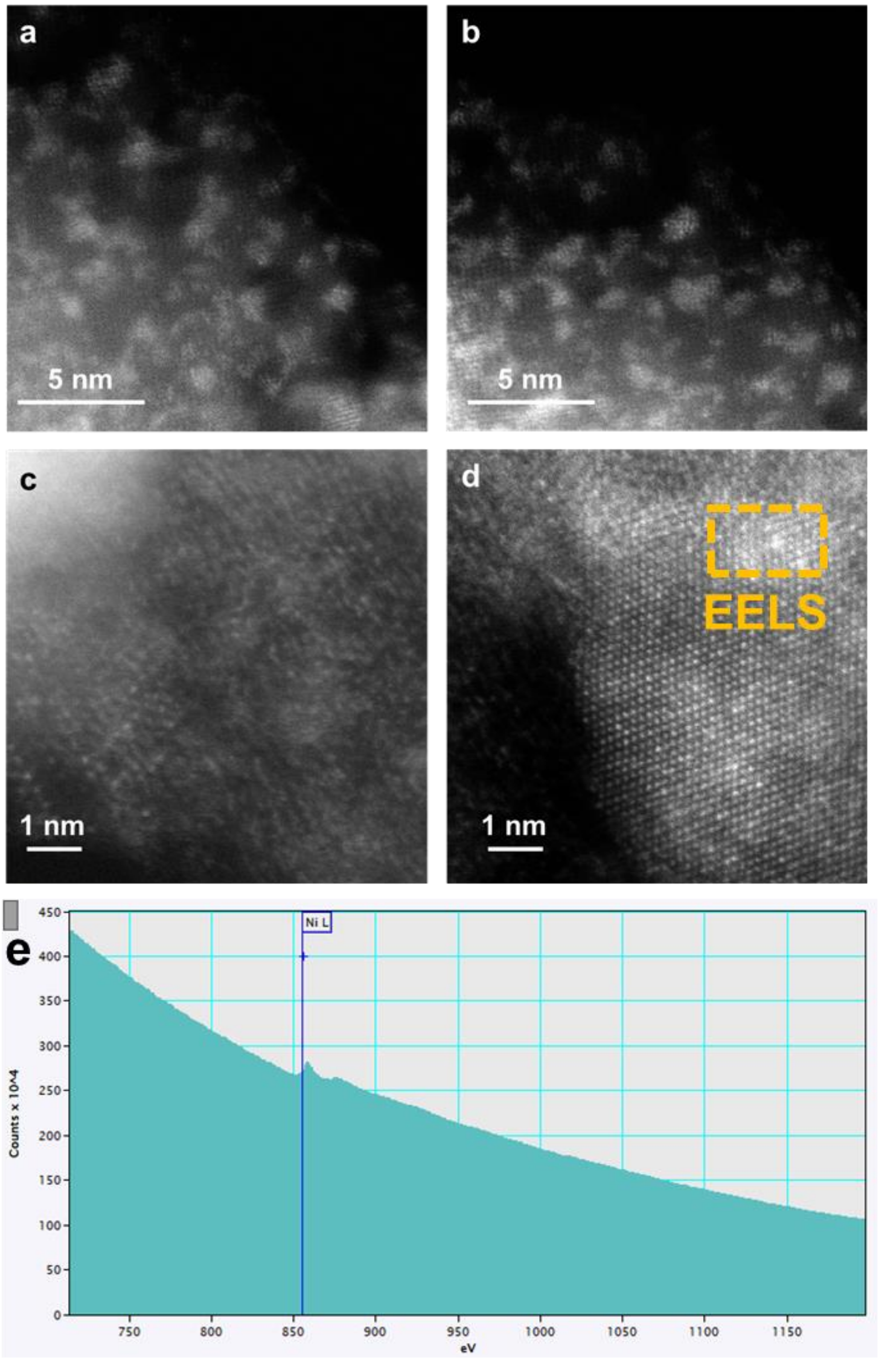

Supplementary Figure 3. a-b TEM. **c-d** high-resolution-TEM. **e** EELS analysis of 1Ni/MTAC on nanoclusters in a bright area of 1Ni/MTAC.

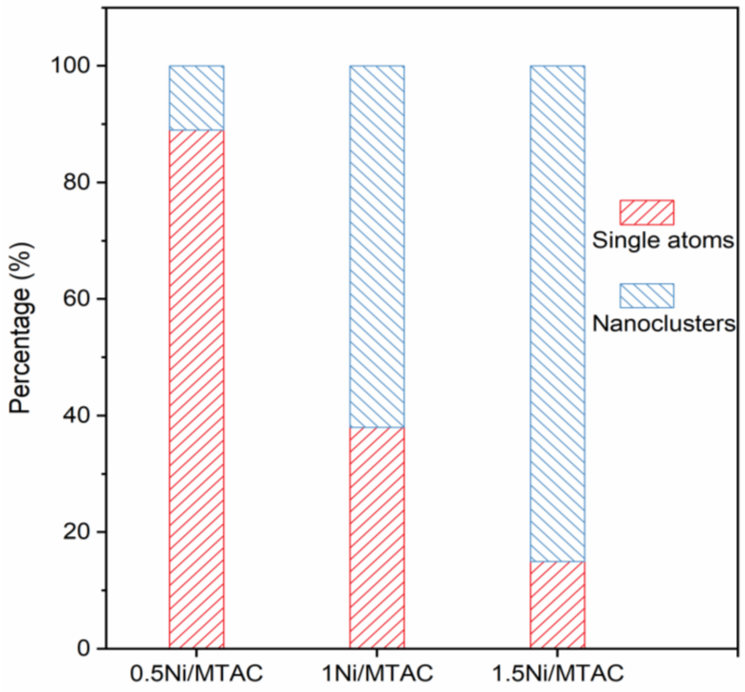

Supplementary Figure 4. Active site distributions of 0.5, 1, and 1.5Ni/MTAC.

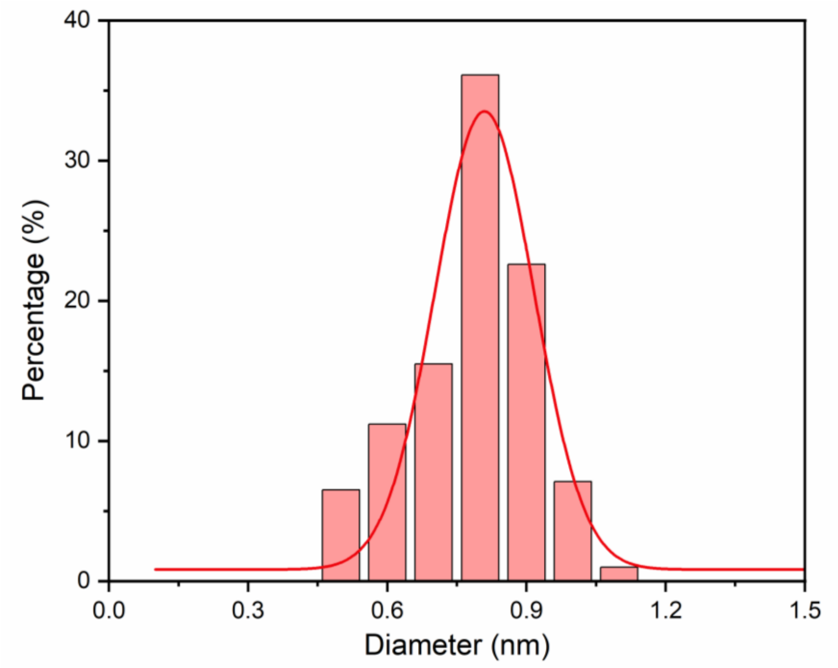

Supplementary Figure 5. Size distribution of 1Ni/MTAC catalyst.

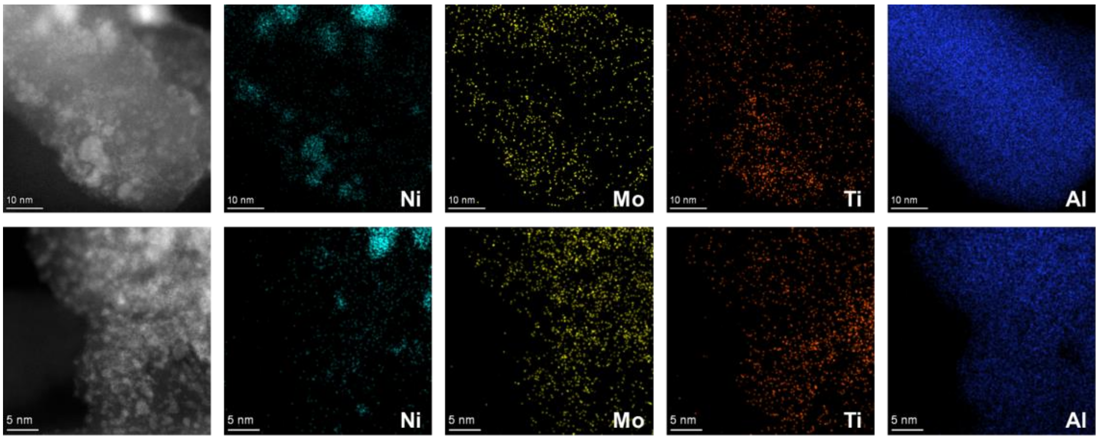

Supplementary Figure 6. EDS mapping results of 1Ni/MTAC sample for Ni, Mo, Ti, and Al elements.

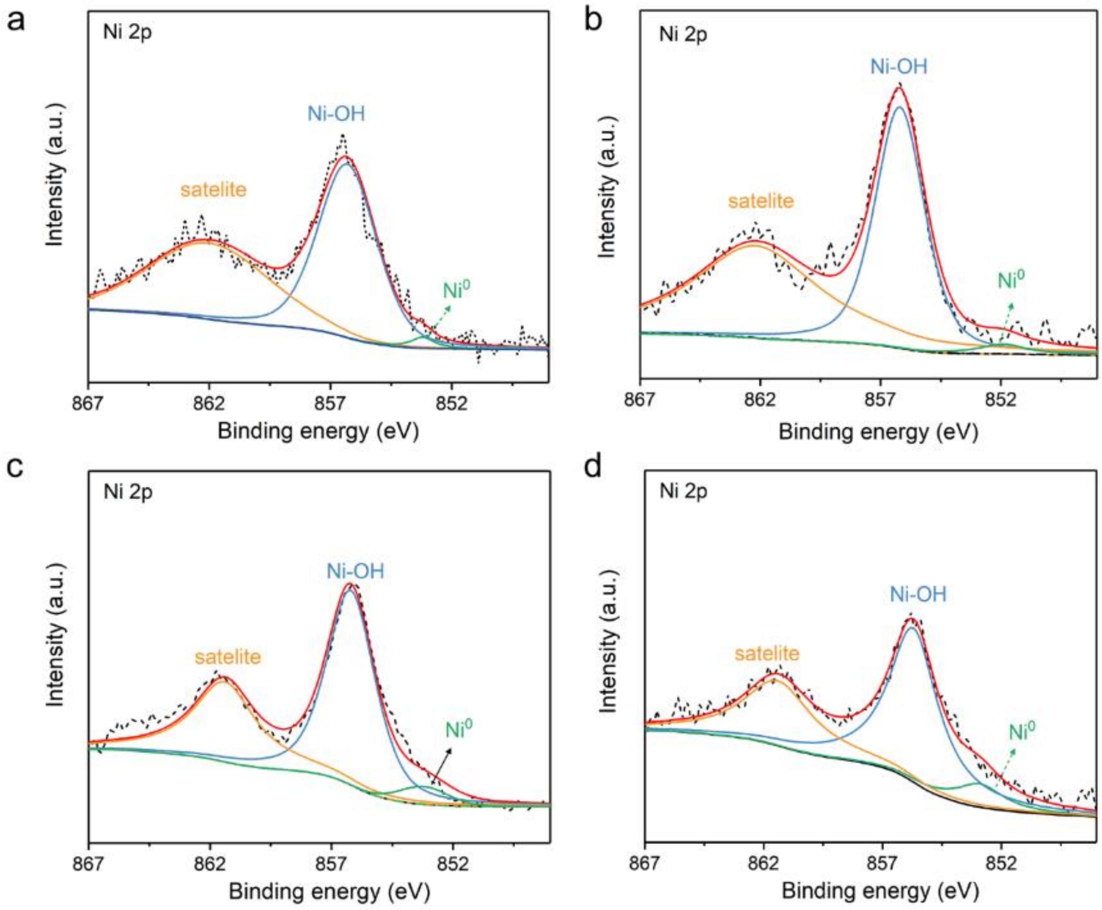

Supplementary Figure 7. The Ni 2*p* XPS spectra of the as-prepared 1Ni/support catalysts. a 1Ni/MTC. **b** 1Ni/Al_2O_3. **c** 1Ni/SiO_2. **d** 1Ni/CeO_2.

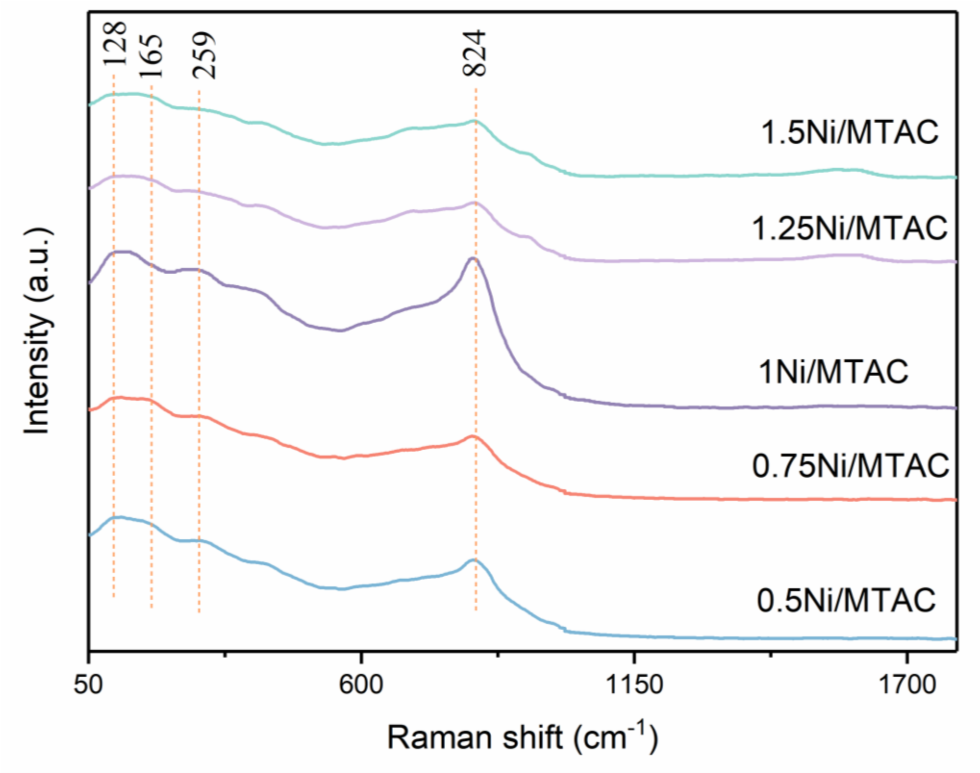

Supplementary Figure 8. Raman spectra of the as-prepared Ni/MTAC catalysts.

For the Raman spectra, the active vibration modes located at 128 cm^{-1} were related to M-A vibrations of the Mo_2TiAlC_2 MAX phase[2]. The band at 165 cm^{-1} can be ascribed to E_g vibrations from Mo and Ti atoms, and the peak at 259 cm^{-1} can be attributed to the E_g vibration of the O atoms, demonstrating the presence of Mo-O structure in MTAC[3]. The sharp band at 824 cm^{-1} can be assigned to hydrated terminal Mo-O and Mo-O-Mo vibrations.

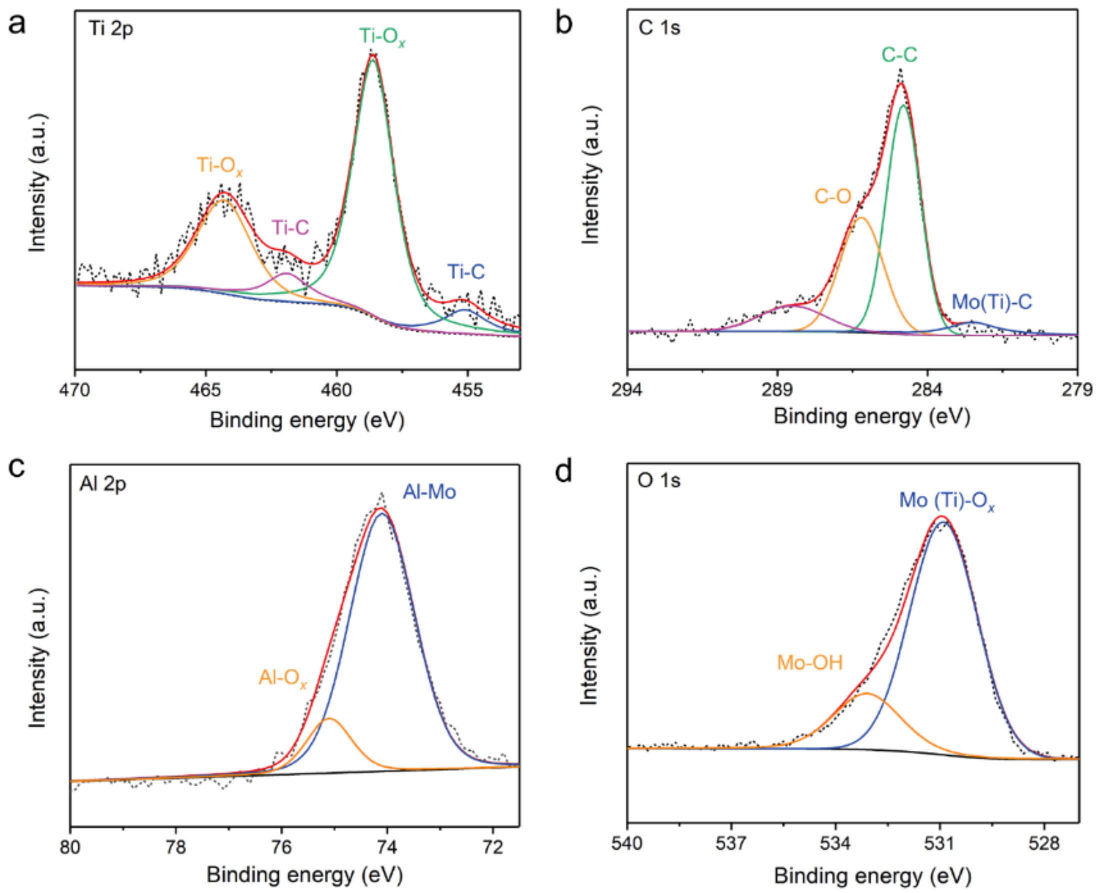

Supplementary Figure 9. XPS spectra of the as-prepared 0.5Ni/MTAC catalyst. a Ti 2*p* spectrum. **b** C 1*s* spectrum. **c** Al 2*p* spectrum. **d** O 1*s* spectrum.

The high-resolution XPS spectra of Ti 2*p* are split into four peaks, at 455.4 eV, 458.9 eV, 461.6 eV, and 464.6 eV, which are assigned to Ti-C, Ti-O_x, Ti-C, and Ti-O_x, respectively. The C 1*s* spectrum is related to binding energies of 283.3 eV, 284.9 eV, 286.2 eV, which are ascribed to Mo(Ti)-C, C-C, and C-O bonds, respectively. The C-O bond is related to the oxygen from the air. Moreover, the XPS result of O 1*s* scans containing two primary peaks at 530.4 and 533.8 eV, which agree with Mo(Ti)-O_x, and Mo-OH, respectively[4]. The peak of Mo(Ti)-O_x is consistent with the result of Mo 3*d* and Ti 2*p* spectra.

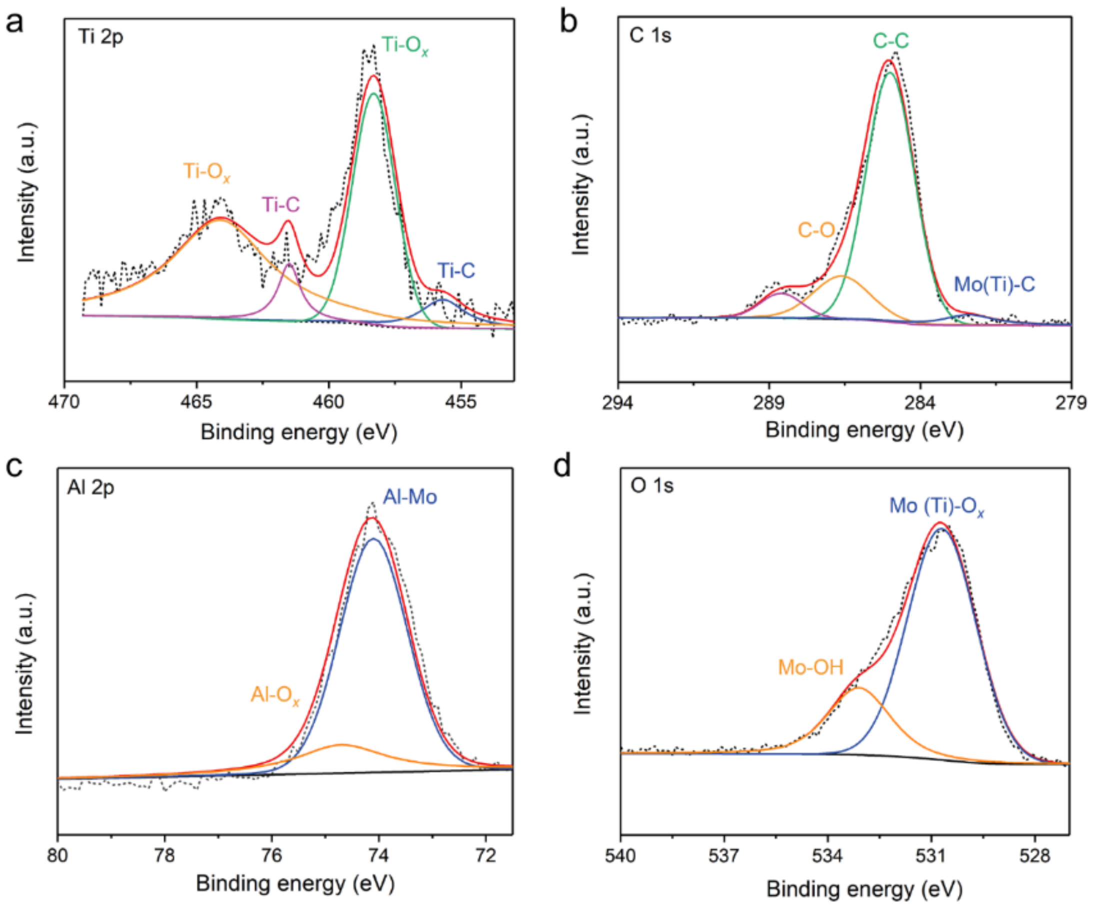

Supplementary Figure 10. XPS spectra of the as-prepared 1Ni/MTAC catalyst. a Ti 2*p* spectrum. **b** C 1*s* spectrum. **c** Al 2*p* spectrum. **d** O 1*s* spectrum.

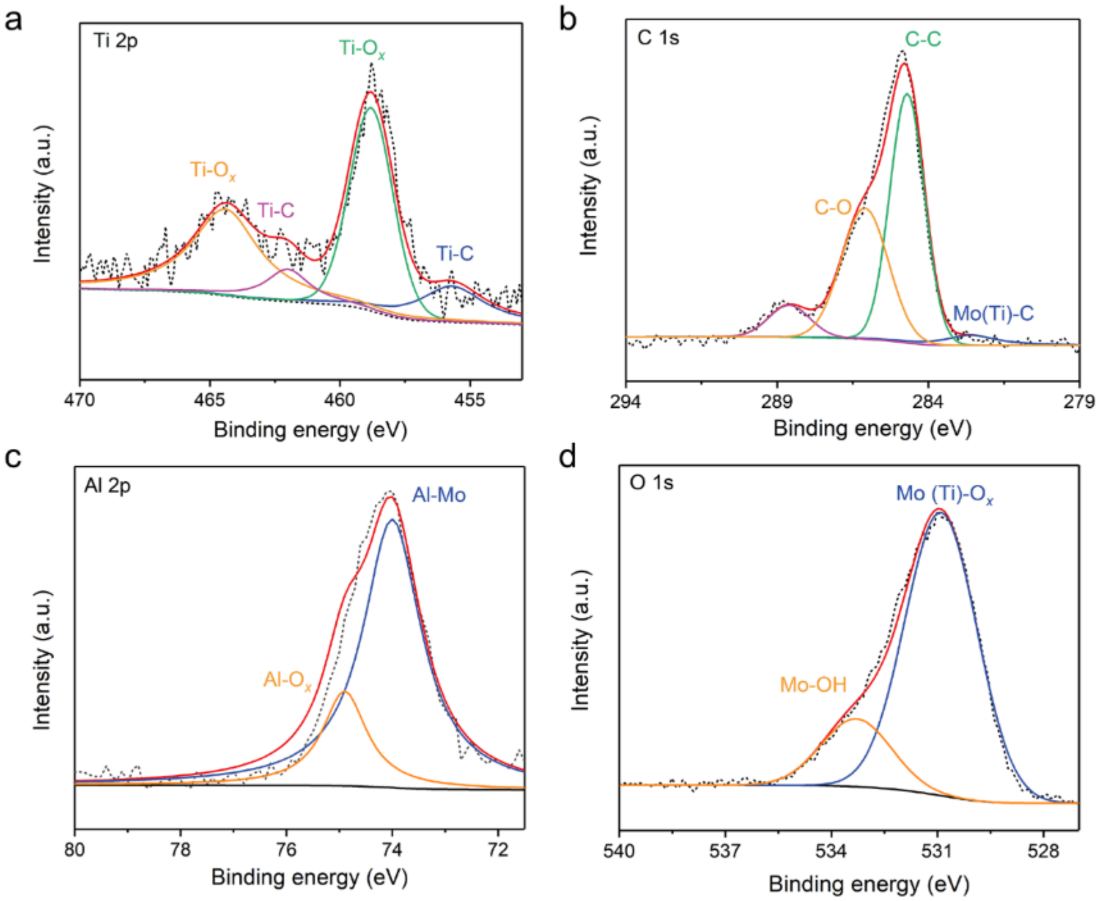

Supplementary Figure 11. XPS spectra of the as-prepared 1Ni/MTAC catalyst. a Ti 2*p* spectrum. **b** C 1*s* spectrum. **c** Al 2*p* spectrum. **d** O 1*s* spectrum.

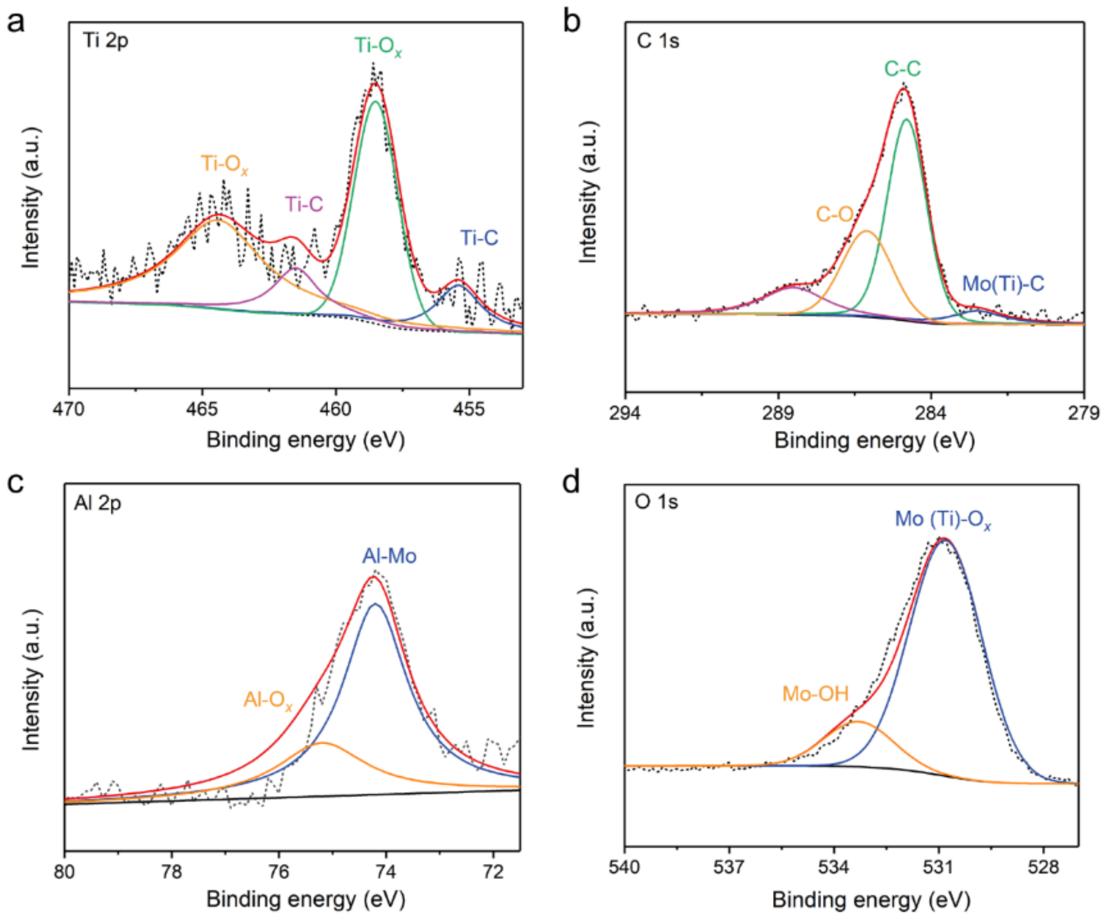

Supplementary Figure 12. XPS spectra of the as-prepared 1.25Ni/MTAC catalyst. a Ti 2*p* spectrum. **b** C 1*s* spectrum. **c** Al 2*p* spectrum. **d** O 1*s* spectrum.

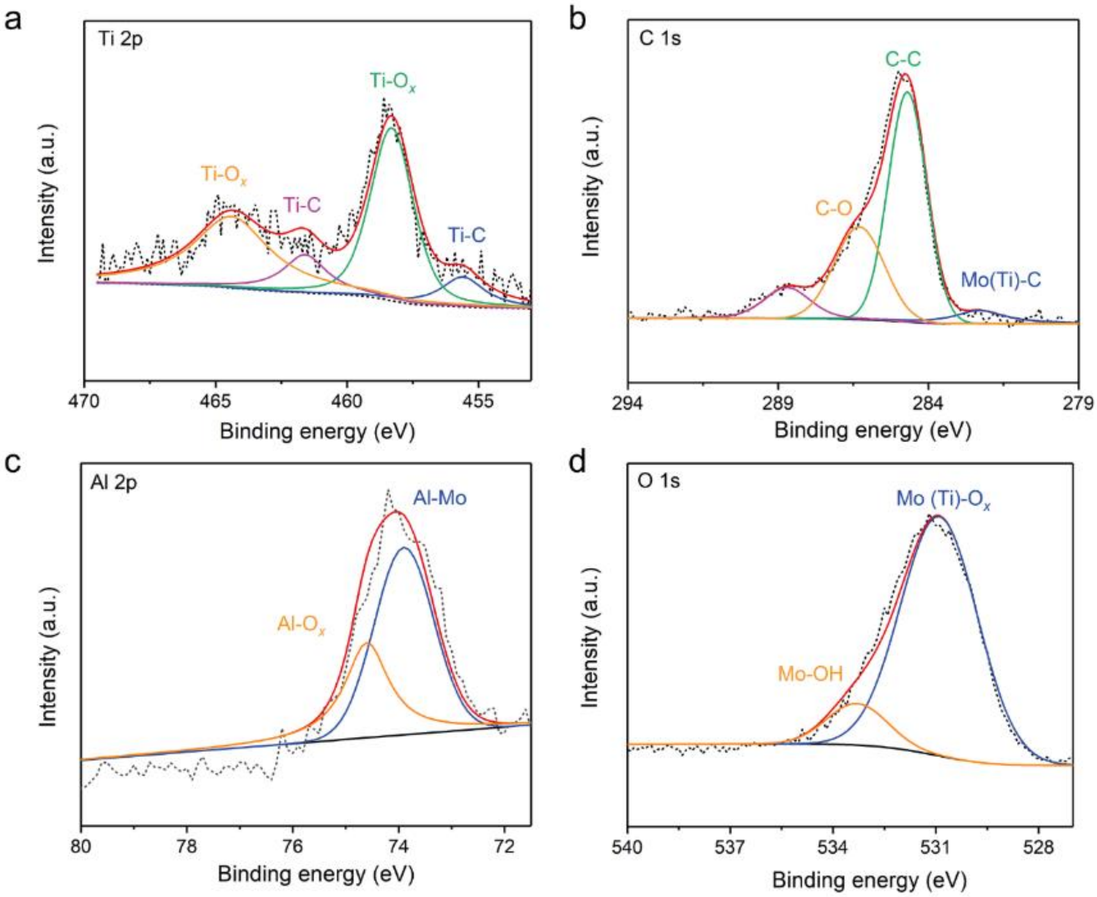

Supplementary Figure 13. XPS spectra of the as-prepared 1.5Ni/MTAC catalyst. a Ti 2*p* spectrum. **b** C 1*s* spectrum. **c** Al 2*p* spectrum. **d** O 1*s* spectrum.

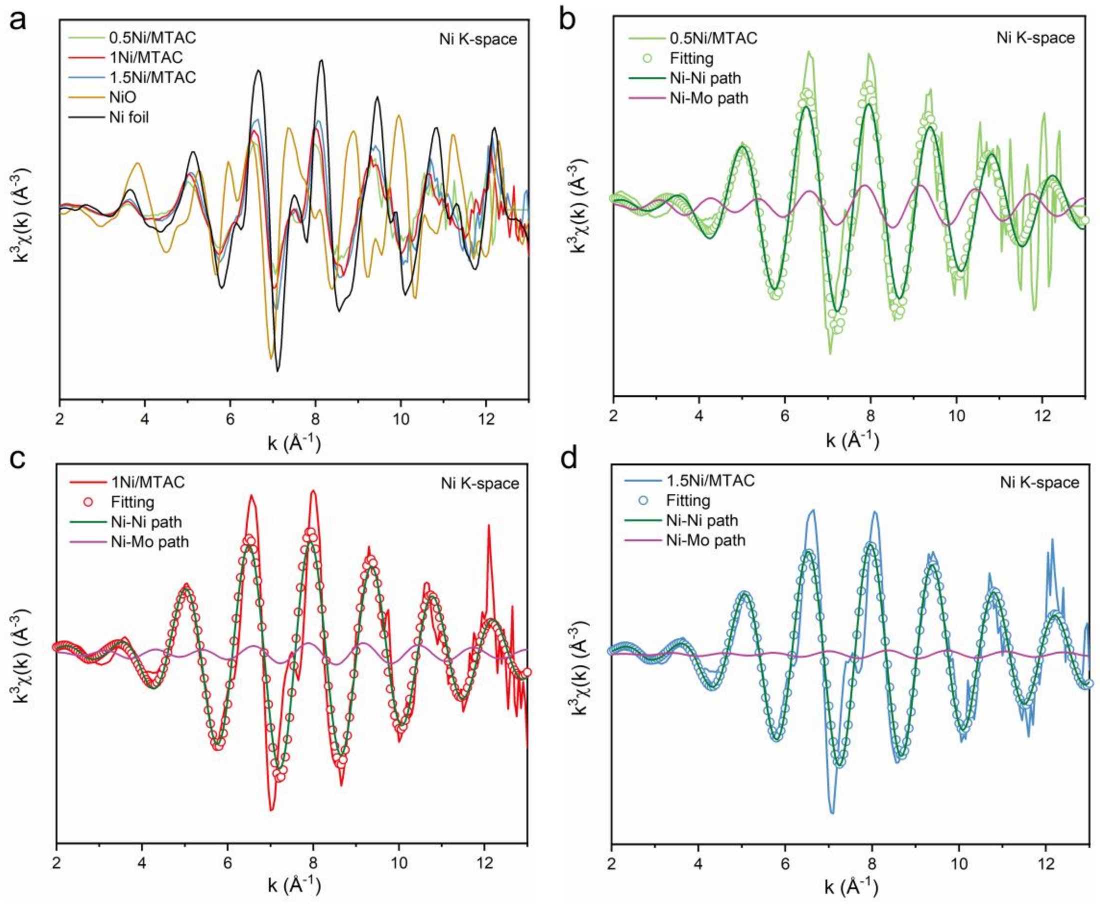

Supplementary Figure 14. XAFS analysis of as-prepared samples. a Fourier transform of Ni K-edge EXAFS spectra in K-space. The fitting results of Ni K-edge Fourier-transform EXAFS spectra on K-space of **b** 0.5Ni/MTAC, **c** 1Ni/MTAC, and **d** 1.5Ni/MTAC.

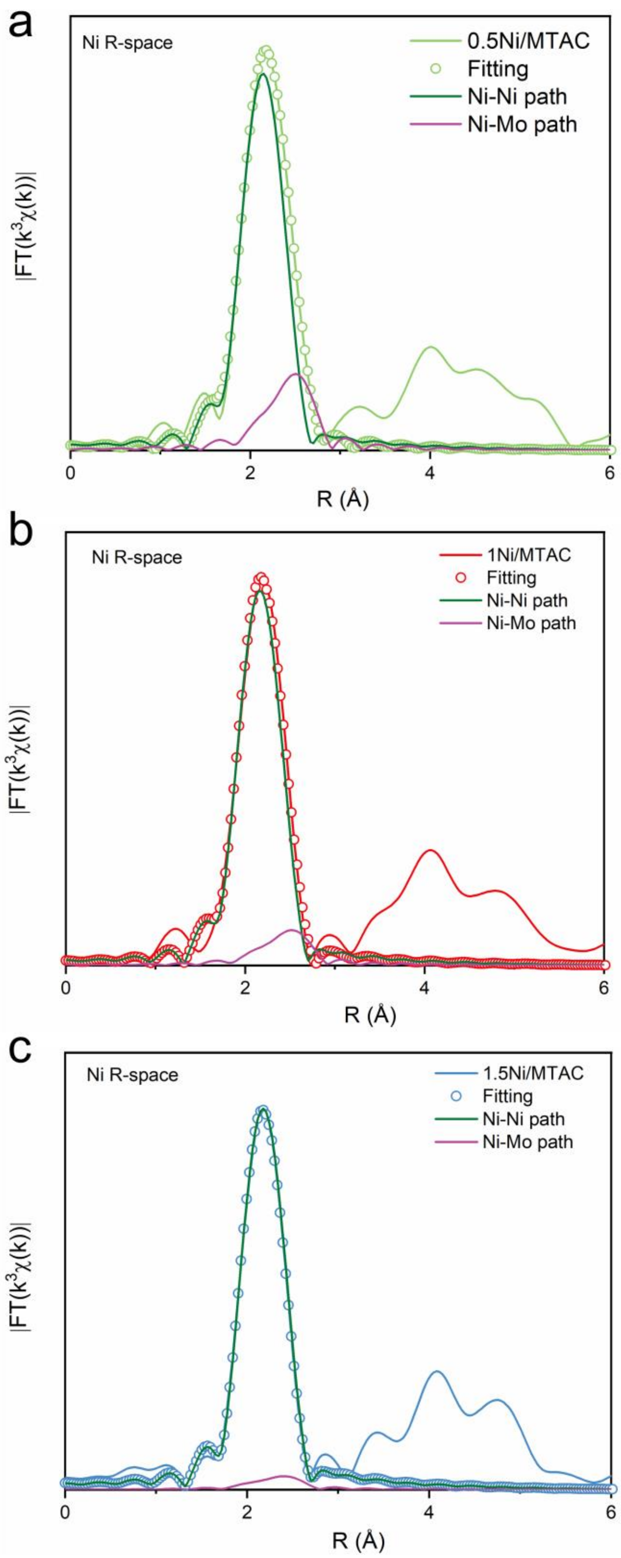

Supplementary Figure 15. The fitting results of Ni K-edge Fourier-transform EXAFS spectra on R-space of **a** 0.5Ni/MTAC, **b** 1Ni/MTAC, and **c** 1.5Ni/MTAC.

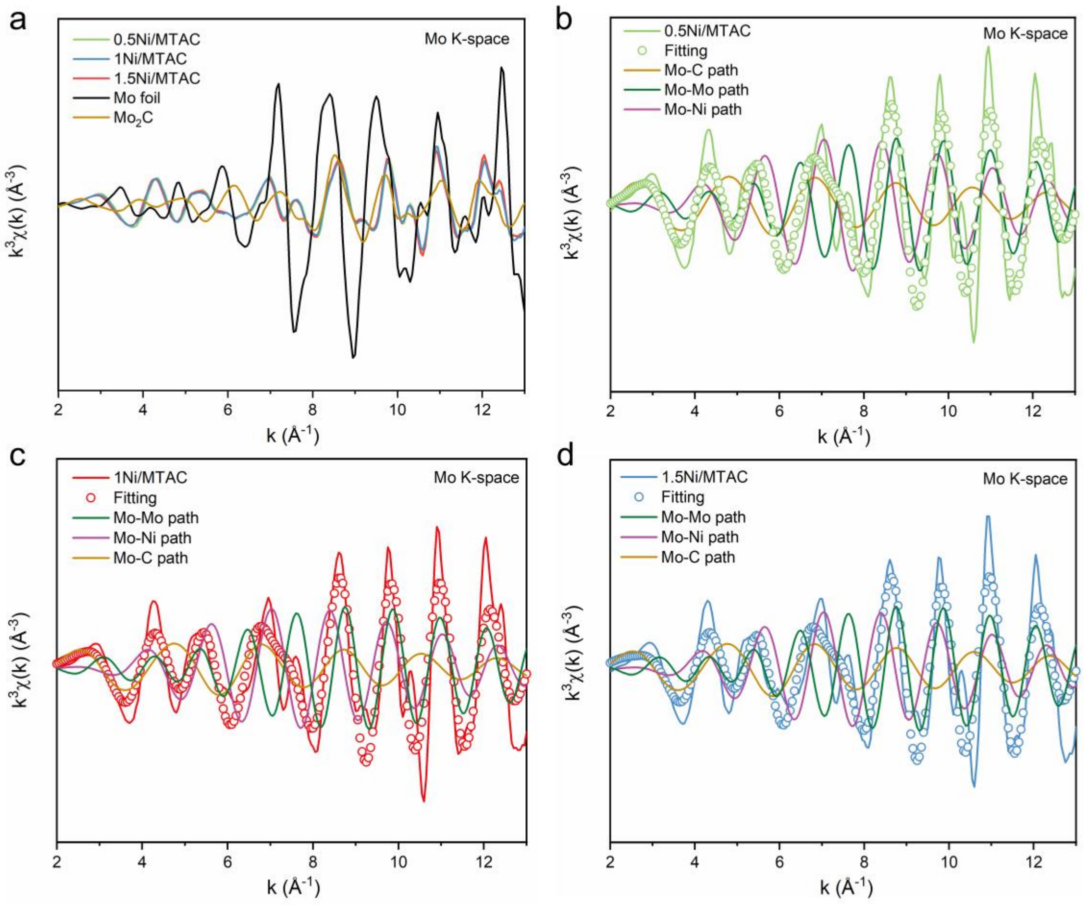

Supplementary Figure 16. XAFS analysis of as-prepared samples. a Fourier transform of Mo K-edge EXAFS spectra in K-space. The fitting results of Mo K-edge Fourier-transform EXAFS spectra on K-space of **b** 0.5Ni/MTAC, **c** 1Ni/MTAC, and **d** 1.5Ni/MTAC.

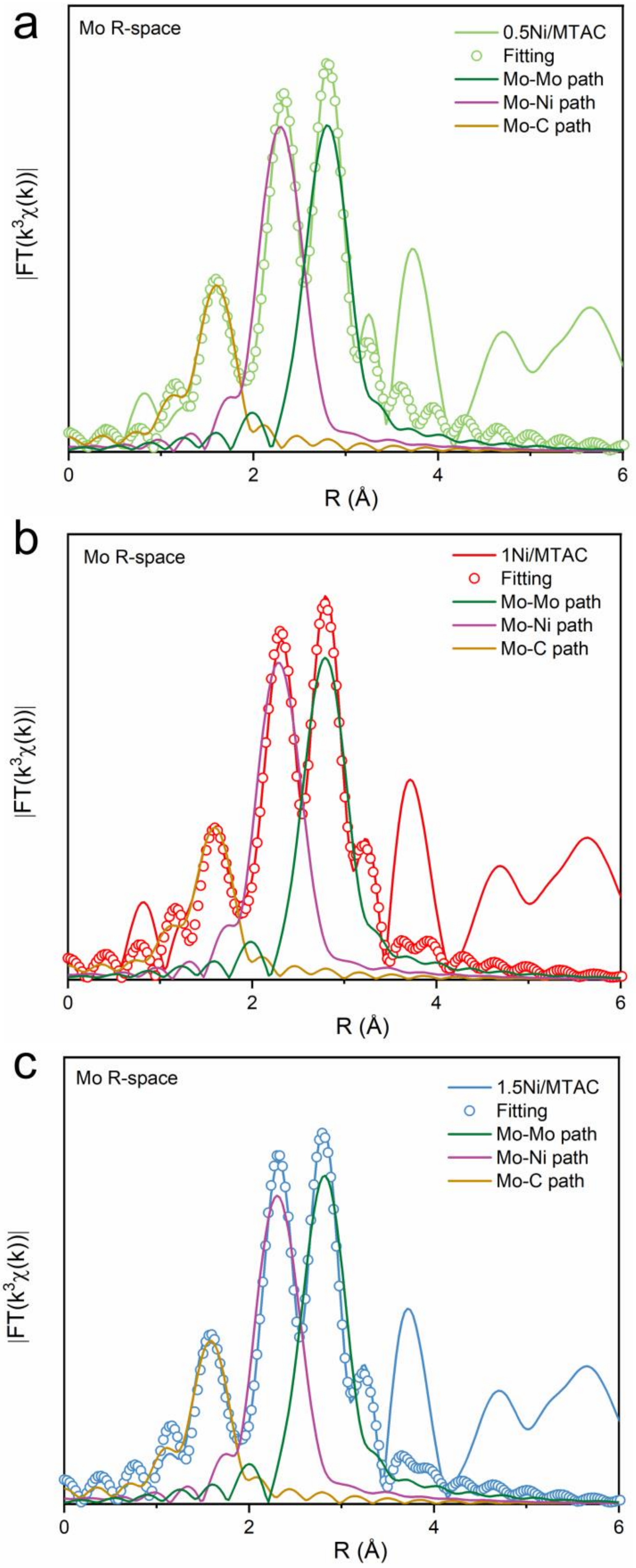

Supplementary Figure 17. The fitting results of Mo K-edge Fourier-transform EXAFS spectra on R-space of **a** 0.5Ni/MTAC, **b** 1Ni/MTAC, and **c** 1.5Ni/MTAC.

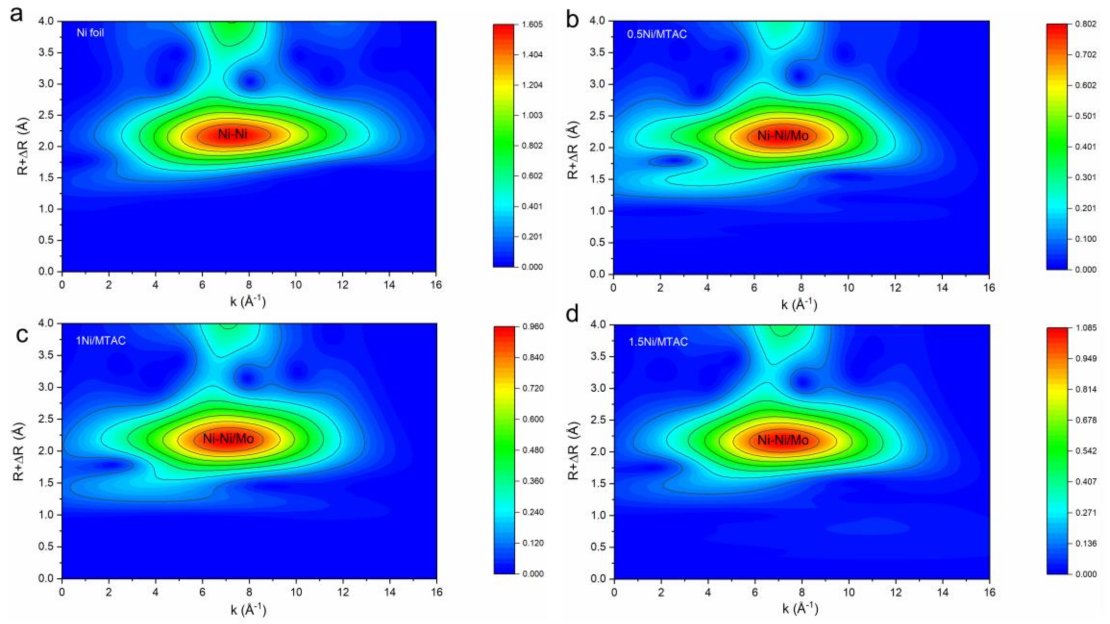

Supplementary Figure 18. Wavelet transformed EXAFS spectra. Ni K-edge EXAFS spectra for **a** Ni foil. **b** 0.5Ni/MTAC. **c** 1Ni/MTAC, and **d** 1.5Ni/MTAC.

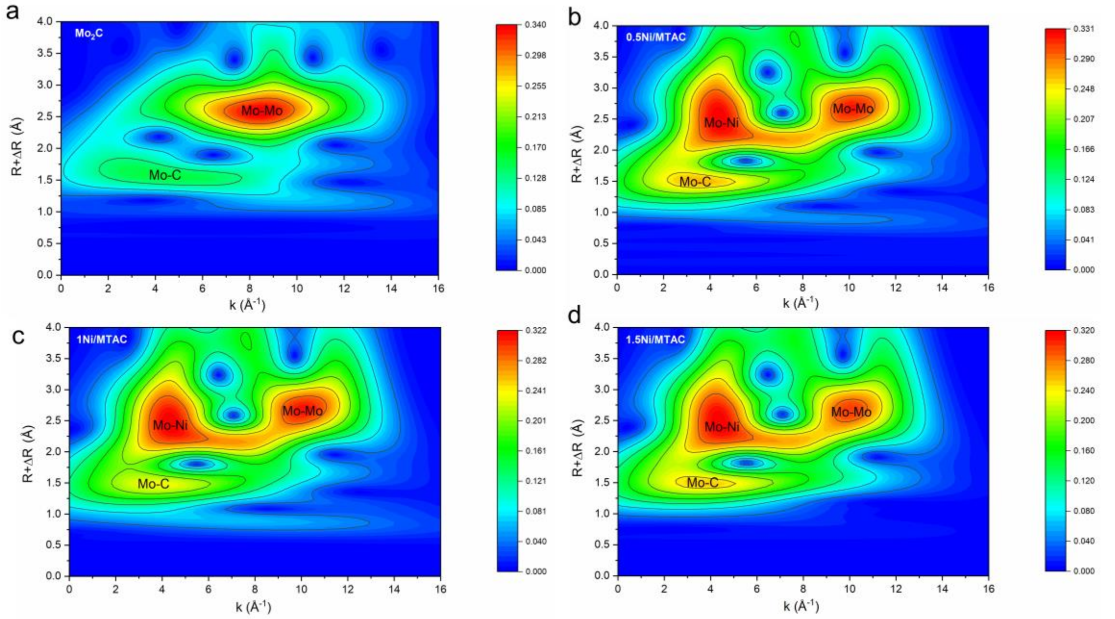

Supplementary Figure 19. Wavelet transformed EXAFS spectra. Mo K-edge EXAFS spectra for **a** Mo_2C. **b** 0.5Ni/MTAC. **c** 1Ni/MTAC, and **d** 1.5Ni/MTAC.

To precisely clarify the atomic dispersion and coordination conditions of Ni and Mo, the wavelet transform of EXAFS spectra were analysed due to its more efficient resolution ability in K-space and radial distance[5], in which the atoms at similar coordination conditions and distances could be distinguished (Supplementary Fig. 16-17)[6, 7]. Ni/MTAC catalysts still displays different maximum intensity with Ni foil and Mo_2C, further implying the formation of the Ni-Mo coordination and the Ni-Mo_n-$Mo_{2-n}TiAlC_2$ structure.

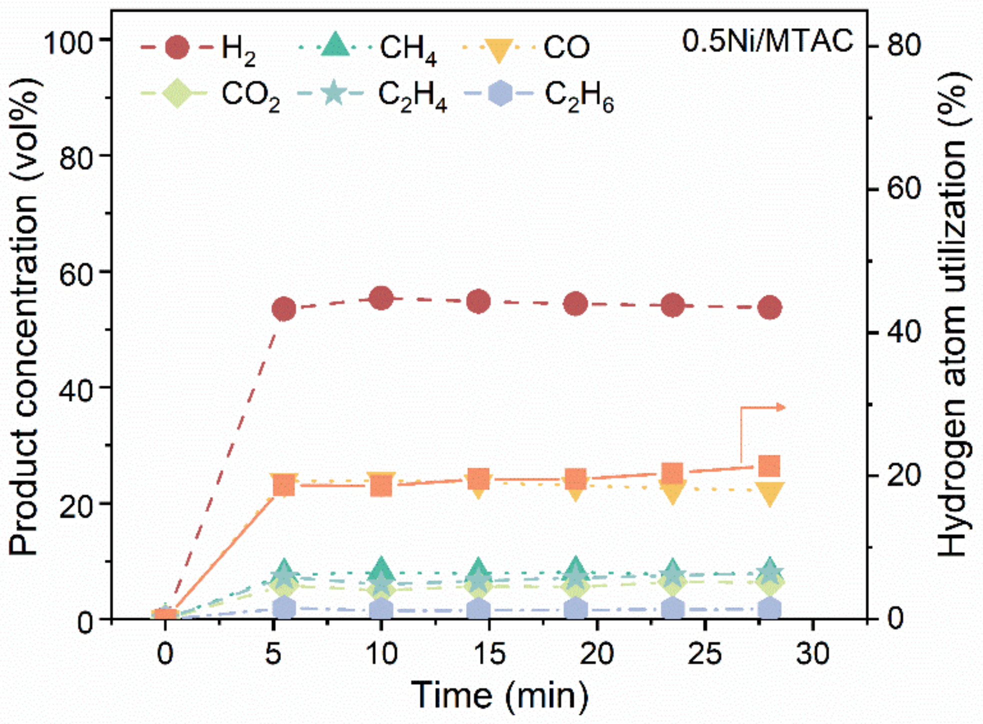

Supplementary Figure 20. SRE performance of the 0.5Ni/MTAC. Activity test conditions: 1 atm, 550 °C, S/E=3, liquid feeding rate 0.005 mL min^{-1}, N_2 flow rate 40 mL min^{-1}.

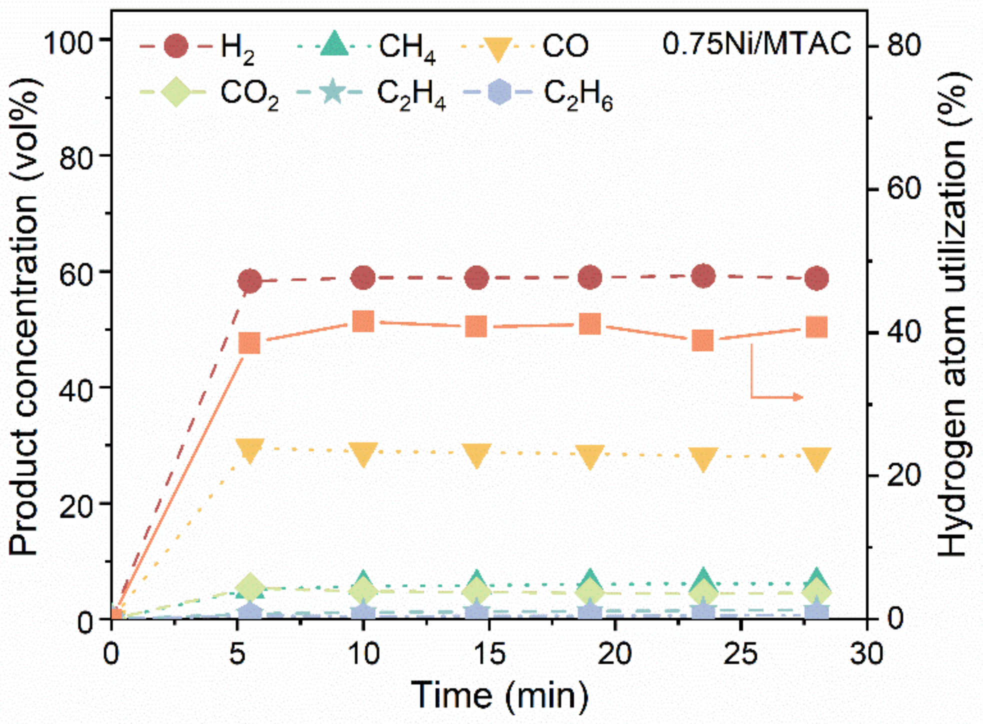

Supplementary Figure 21. SRE performance of the 0.75Ni/MTAC. Activity test conditions: 1 atm, 550 °C, S/E=3, liquid feeding rate 0.005 mL min^{-1}, N_2 flow rate 40 mL min^{-1}.

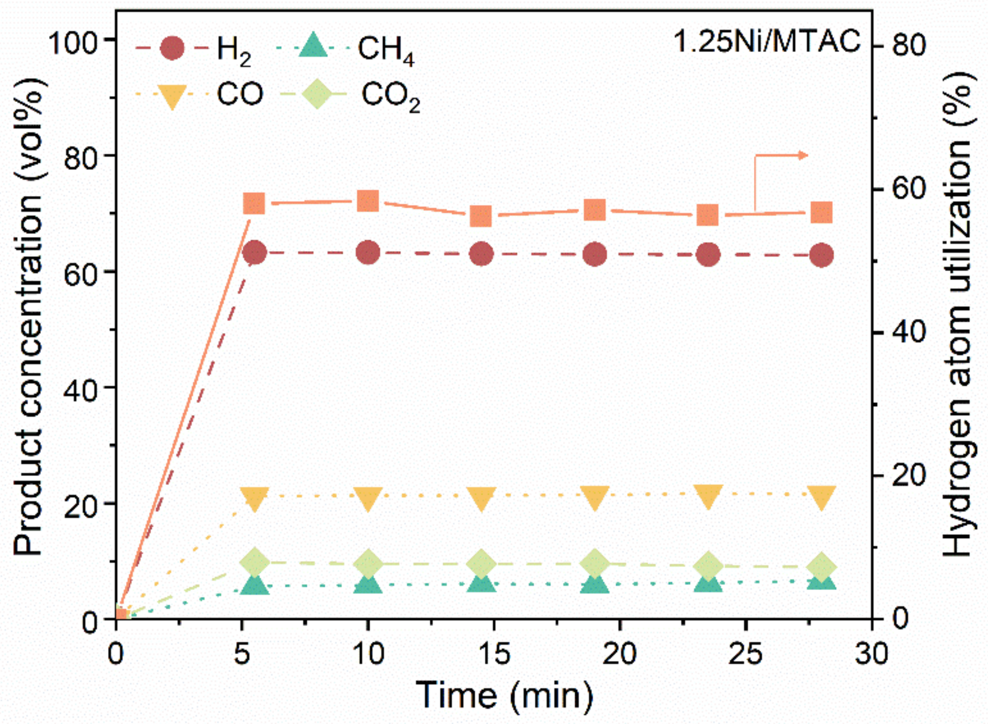

Supplementary Figure 22. SRE performance of the 1.25Ni/MTAC. Activity test conditions: 1 atm, 550 °C, S/E=3, liquid feeding rate 0.005 mL min^{-1}, N_2 flow rate 40 mL min^{-1}.

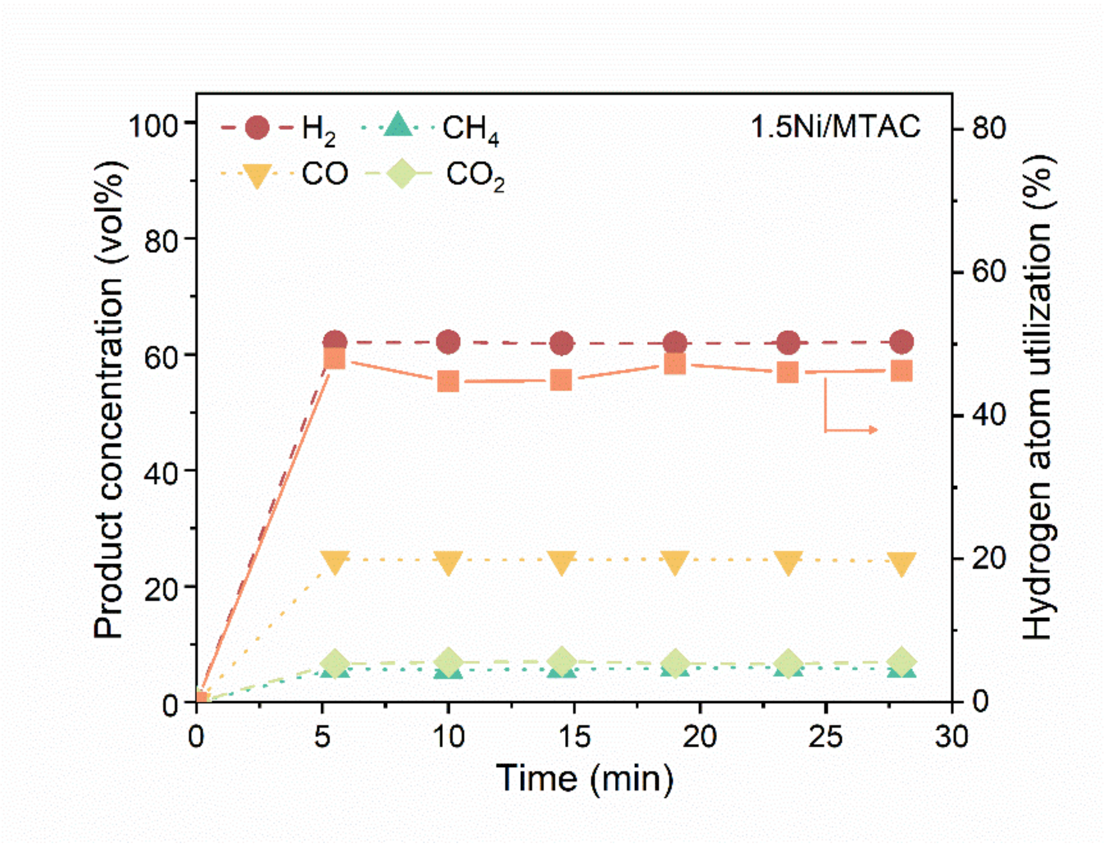

Supplementary Figure 23. SRE performance of the 1.5Ni/MTAC. Activity test conditions: 1 atm, 550 °C, S/E=3, liquid feeding rate 0.005 mL min^{-1}, N_2 flow rate 40 mL min^{-1}.

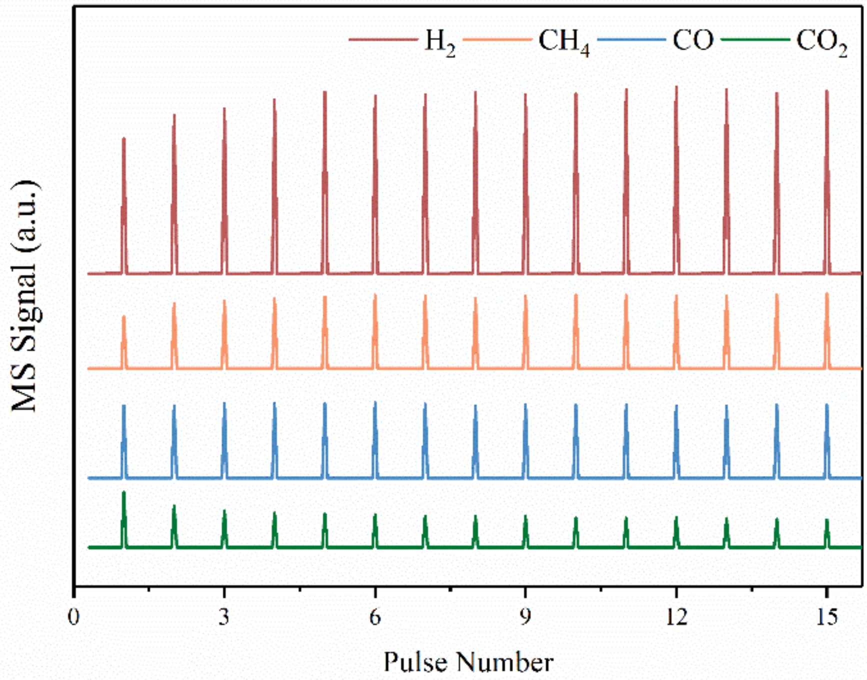

Supplementary Figure 24. MS signals from the operando pulse experiment of ethanol and water (S/E=3) over 1Ni/MTAC catalyst at 550 °C.

From the perspective of active site estimation, the assumptions are as follows: It is considered that i) a same weight of catalyst is added (1.000 g); ii) the proportions of single atoms are in accordance with the statistical data from TEM (89%, 38%, and 15%); iii) there are around 50 atoms for a Ni nanoclusters with an average size of ~0.6 nm.

Based on the above assumptions, <u>for 0.5Ni/MTAC sample</u>, the approximately numbers of cluster sites would be around 8.81×10^{17}, the approximately numbers of single-atom sites would be 7.13×10^{18}, the approximately numbers of Ni in nanoclusters would be 4.41×10^{19}, and the approximately numbers of Ni in single atoms would be 7.13×10^{18}, total active site number would be **<u>8.01×10^{18}</u>**; <u>for 1Ni/MTAC sample</u>, the approximately numbers of cluster sites would be 1.27×10^{18}, the approximately numbers of single-atom sites would be 3.90×10^{19}, the approximately numbers of Ni in nanoclusters would be 6.36×10^{19}, and the approximately numbers of Ni in single atoms would be 3.90×10^{19}, total active site number would be **<u>40.27×10^{18}</u>**; <u>for 1.5Ni/MTAC sample</u>, the approximately numbers of cluster sites would be 2.62×10^{18}, the approximately numbers of single-atom sites would be 2.31×10^{19}, the approximately numbers of Ni in nanoclusters would be 1.31×10^{20}, and the approximately numbers of Ni in single atoms would be 2.31×10^{19}, total active site number would be **<u>25.72×10^{18}</u>**. Therefore, we suppose that the calculation of the total active site number can to some extent explain the better performance of 1Ni/MTAC sample.

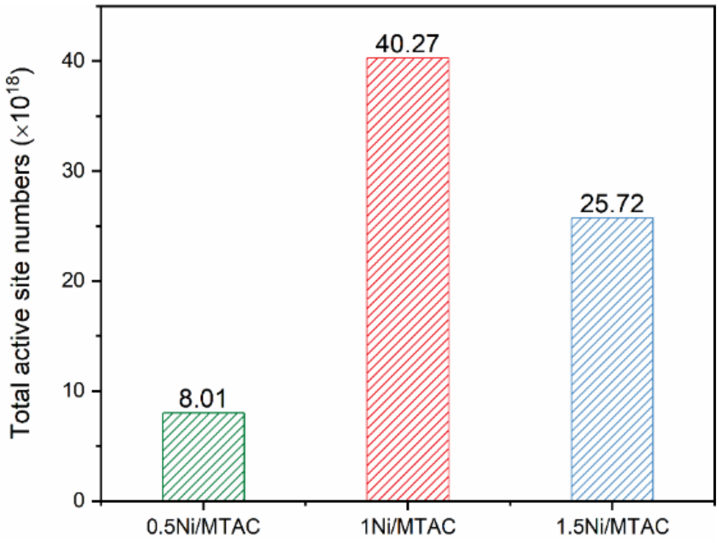

Supplementary Figure 25. Estimated total active site numbers of 0.5Ni/MTAC, 1Ni/MTAC, and 1.5Ni/MTAC catalysts.

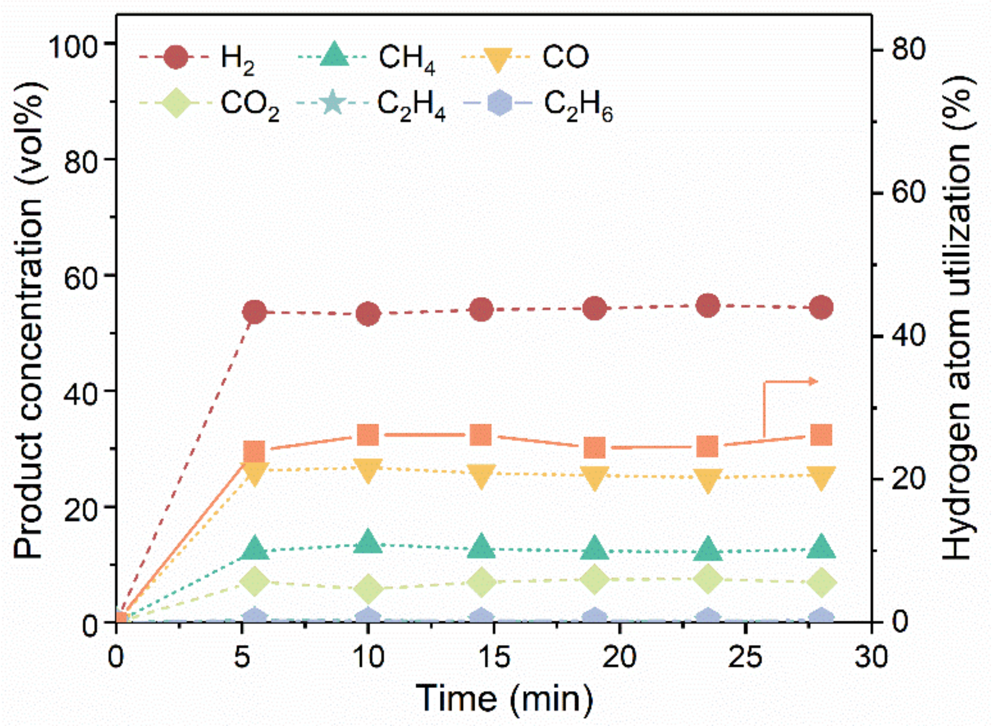

Supplementary Figure 26. SRE performance of 1Ni/MTAC at 450 °C. Activity test conditions: 1 atm, S/E=3, liquid feeding rate 0.005 mL min^{-1}, N_2 flow rate 40 mL min^{-1}.

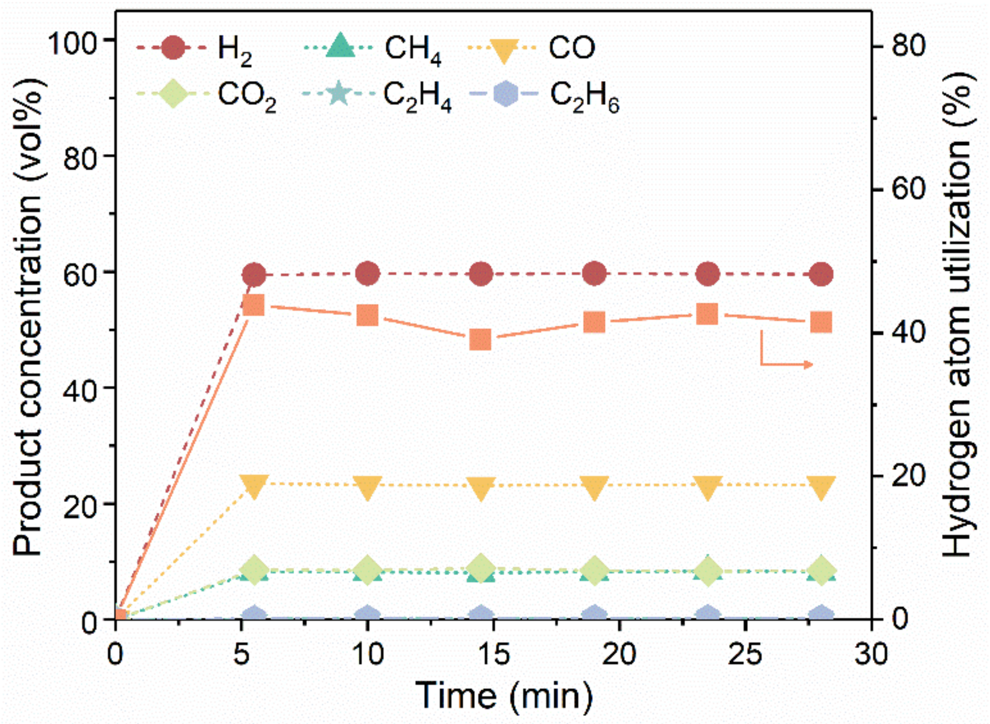

Supplementary Figure 27. SRE performance of 1Ni/MTAC at 500 °C. Activity test conditions: 1 atm, S/E=3, liquid feeding rate 0.005 mL min^{-1}, N_2 flow rate 40 mL min^{-1}.

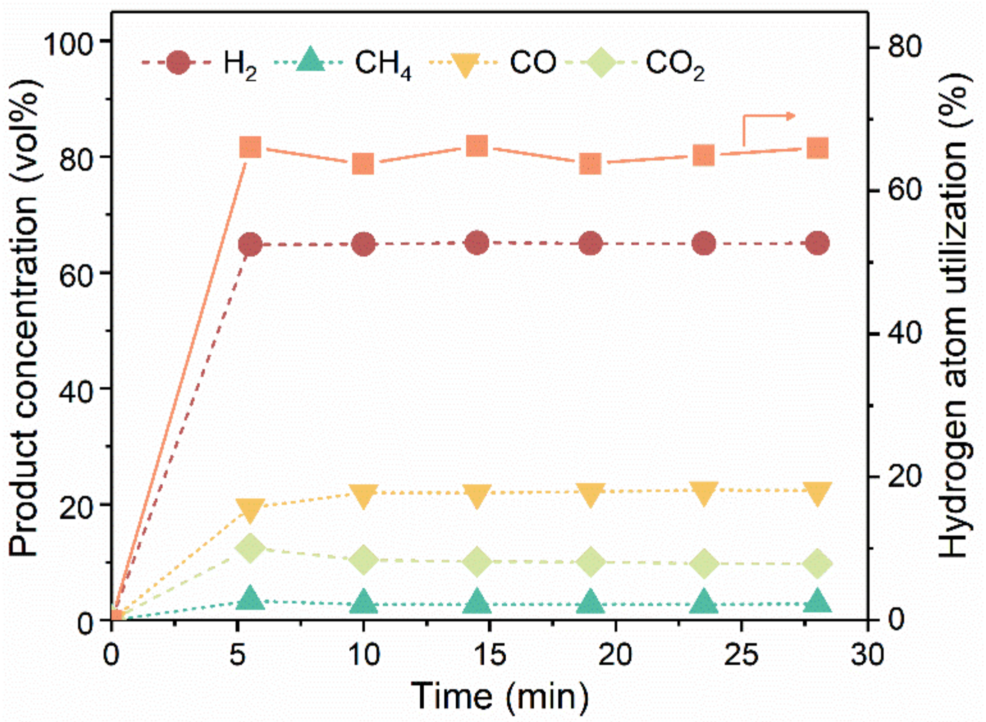

Supplementary Figure 28. SRE performance of 1Ni/MTAC at 600 °C. Activity test conditions: 1 atm, S/E=3, liquid feeding rate 0.005 mL min^{-1}, N_2 flow rate 40 mL min^{-1}.

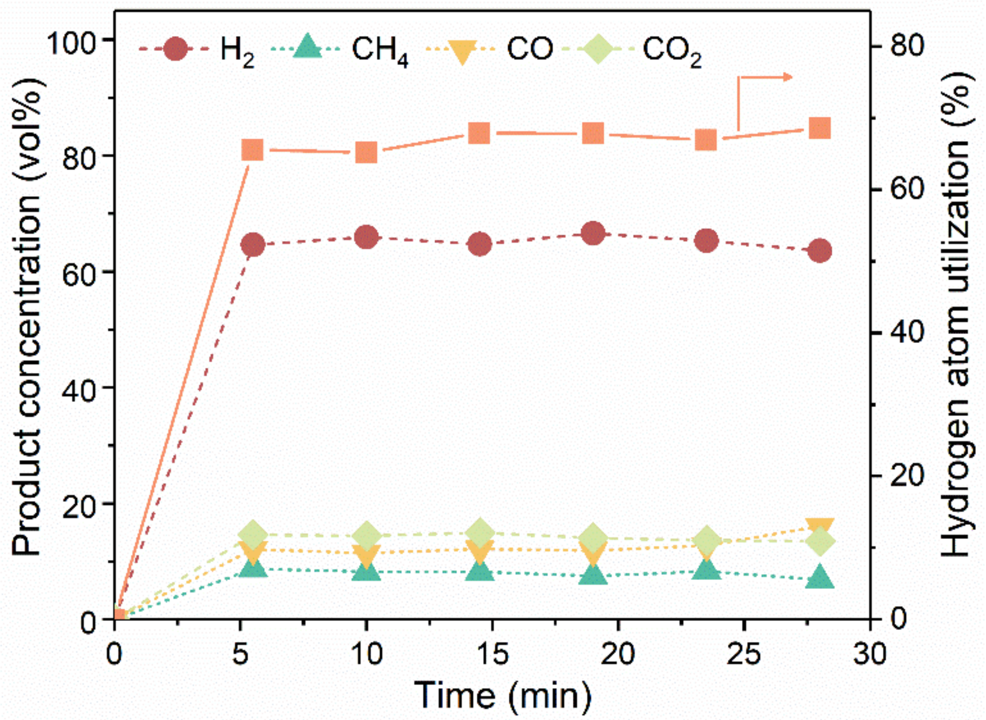

Supplementary Figure 29. SRE performance of 1Ni/MTAC at GHSV values for 2890 h^{-1}. Activity test conditions: 1 atm, 550 °C, S/E=3, liquid feeding rate 0.005 mL min^{-1}, N_2 flow rate 30 mL min^{-1}.

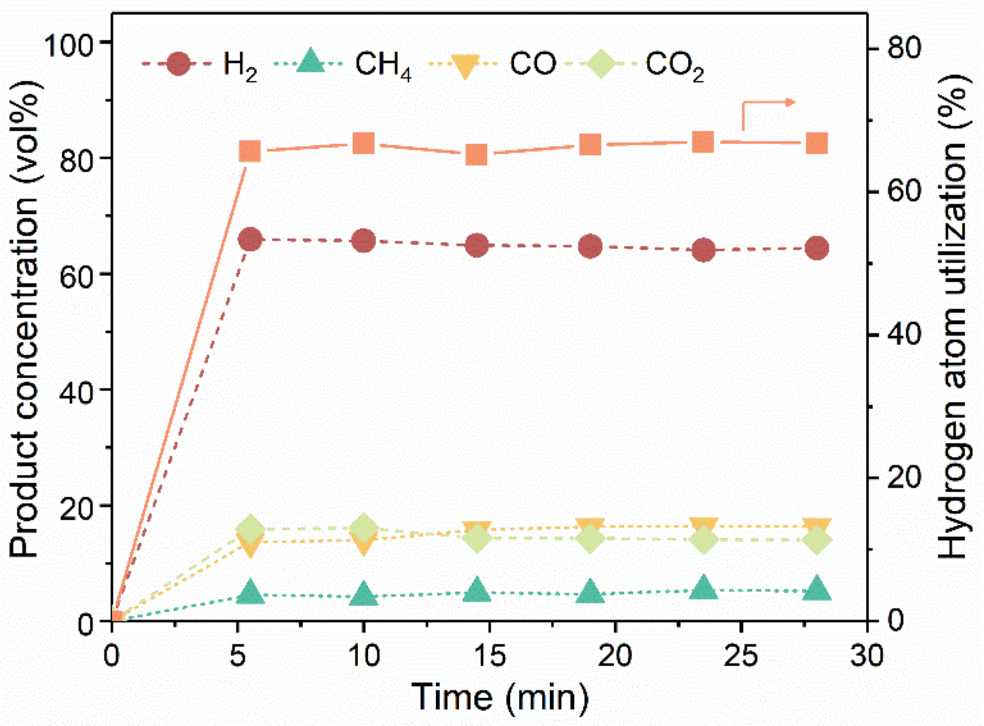

Supplementary Figure 30. SRE performance of 1Ni/MTAC at GHSV values for 3316 h^{-1}. Activity test conditions: 1 atm, 550 °C, S/E=3, liquid feeding rate 0.005 mL min^{-1}, N_2 flow rate 35 mL min^{-1}.

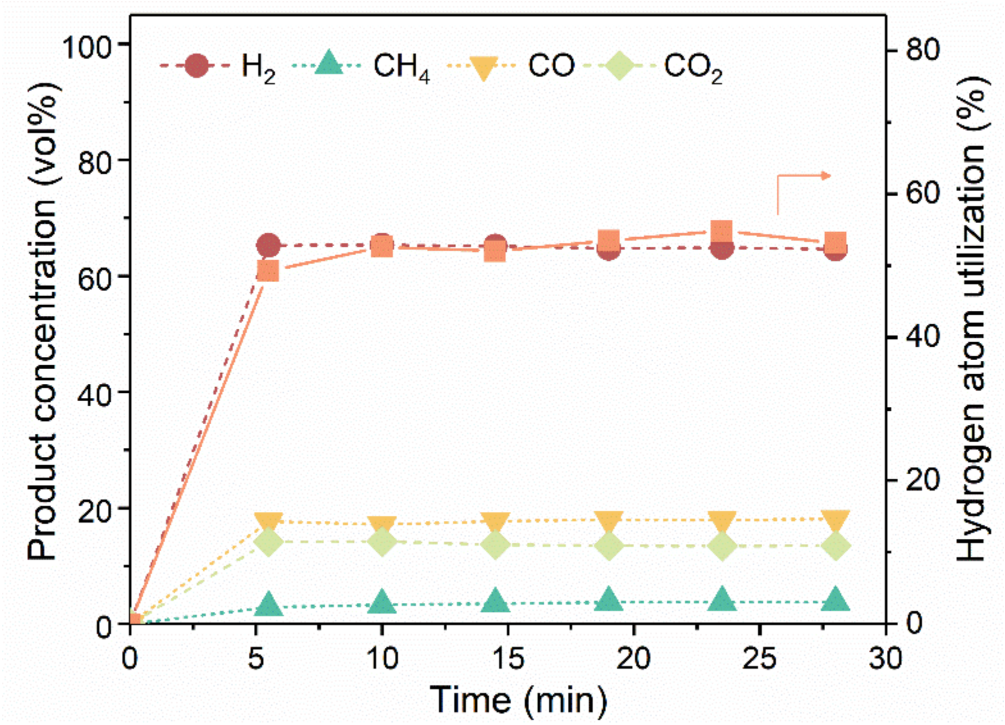

Supplementary Figure 31. SRE performance of 1Ni/MTAC at GHSV values for 4168 h^{-1}. Activity test conditions: 1 atm, 550 °C, S/E=3, liquid feeding rate 0.005 mL min^{-1}, N_2 flow rate 45 mL min^{-1}.

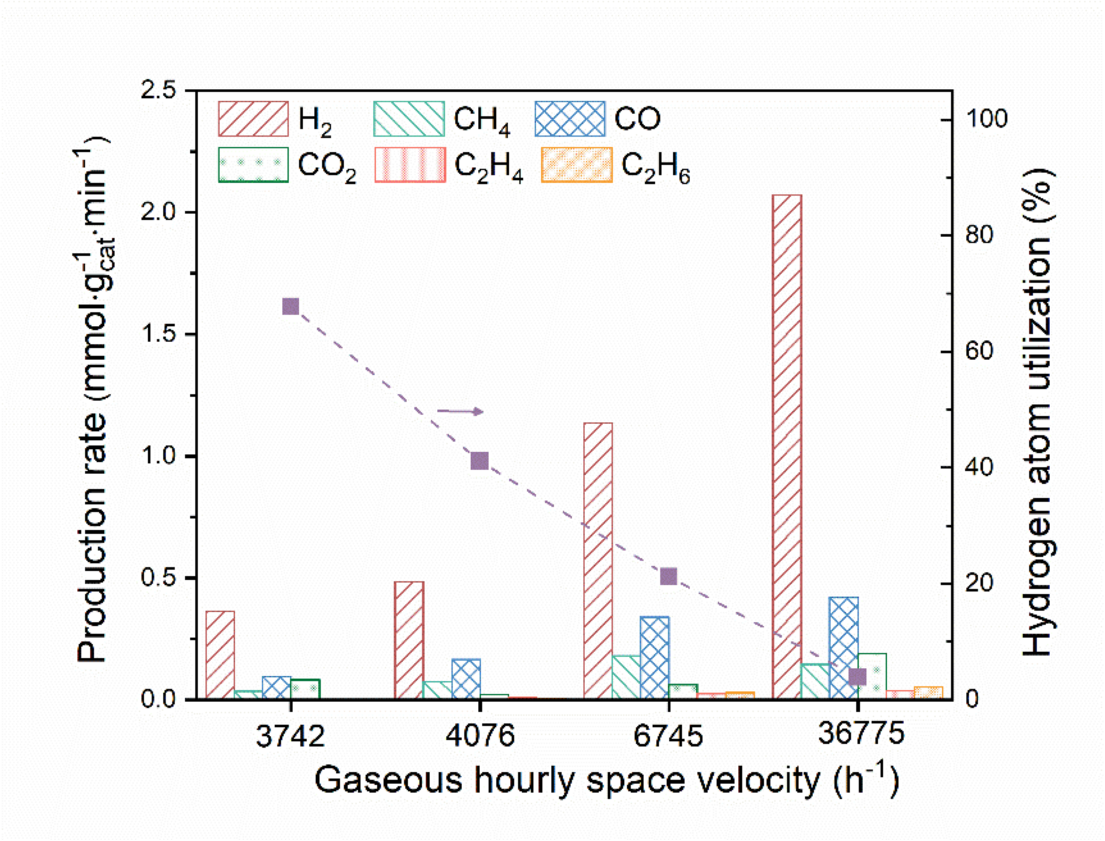

Supplementary Figure 32. SRE performance of 1Ni/MTAC at different liquid feeding rates. **(**The GHSV values for 3742 h^{-1}**,** 4076 h^{-1}, 6745 h^{-1}, and 36775 h^{-1} are 0.005, 0.01, 0.05, and 0.5 liquid feeding rate mL·min^{-1}) Activity test conditions: 1 atm, 550 °C, S/E=3, N_2 flow rate 40 mL min^{-1}.

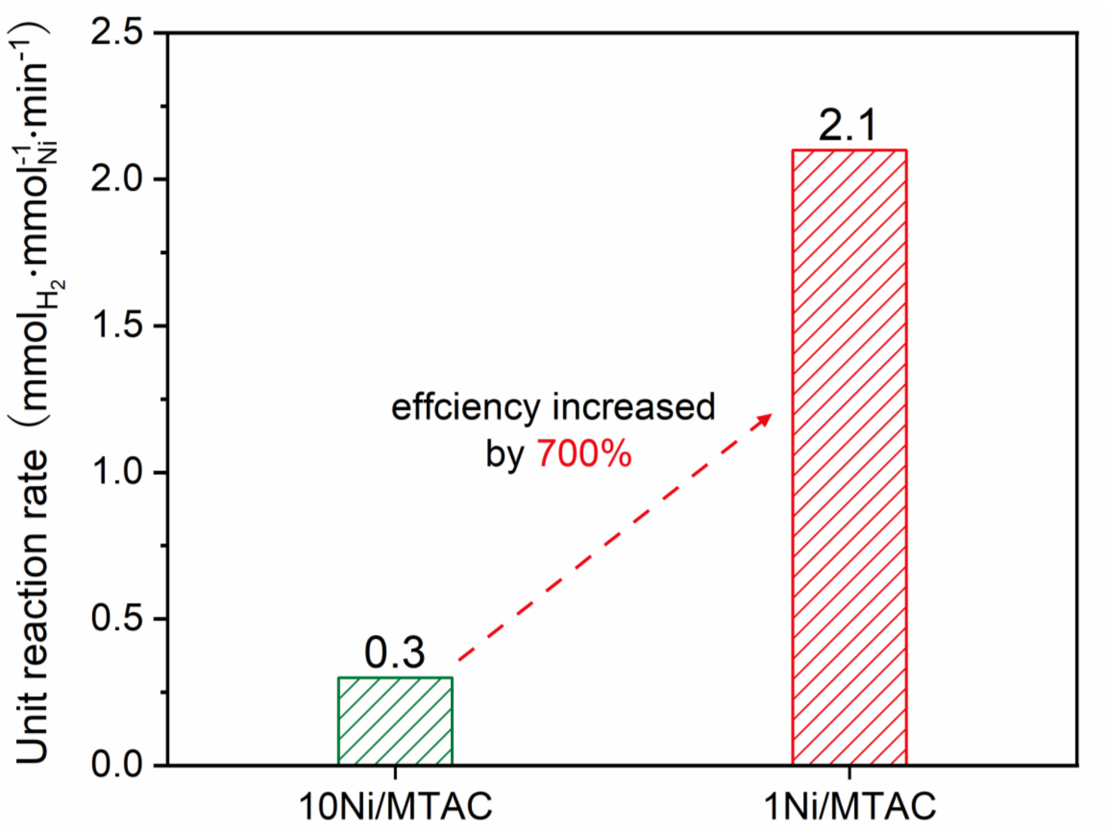

Supplementary Figure 33. Unit reaction rate of 1Ni/MTAC and 10Ni/MTAC. Activity test conditions: 1 atm, 550 °C, S/E=3, liquid feeding rate 0.005 mL min^{-1}, N_2 flow rate 40 mL min^{-1}.

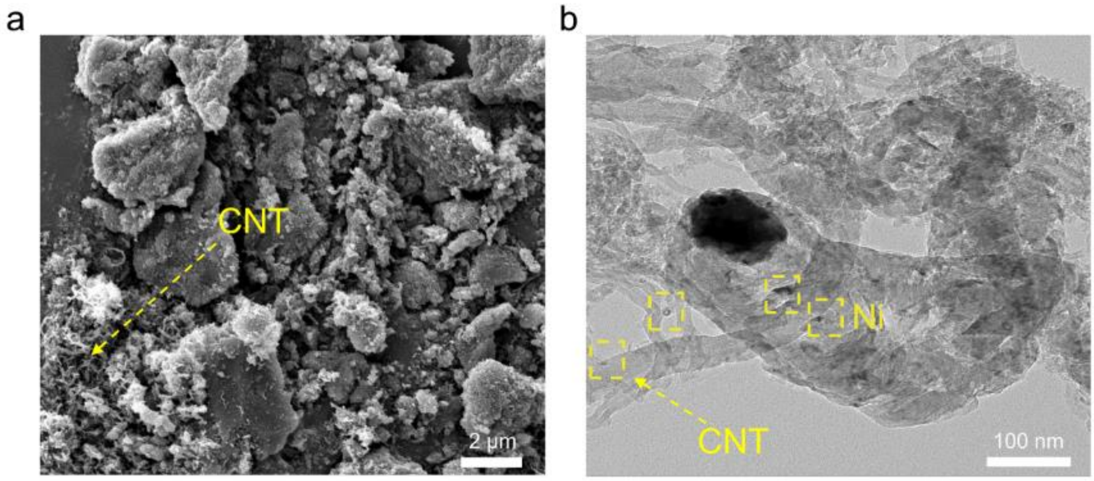

Supplementary Figure 34. Morphology characterization of the 120 h reacted 1Ni/MTAC catalyst. a SEM image. **b** TEM image.

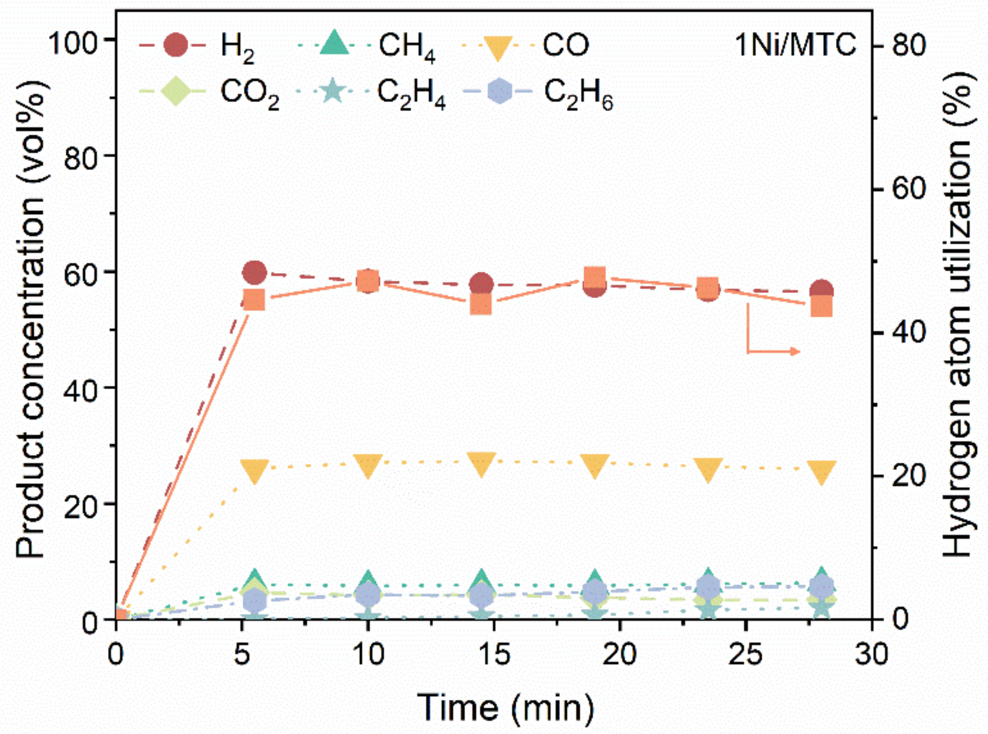

Supplementary Figure 35. SRE performance of 1Ni/MTC. Activity test conditions: 1 atm, 550 °C, S/E=3, liquid feeding rate 0.005 mL min^{-1}, N_2 flow rate 40 mL min^{-1}.

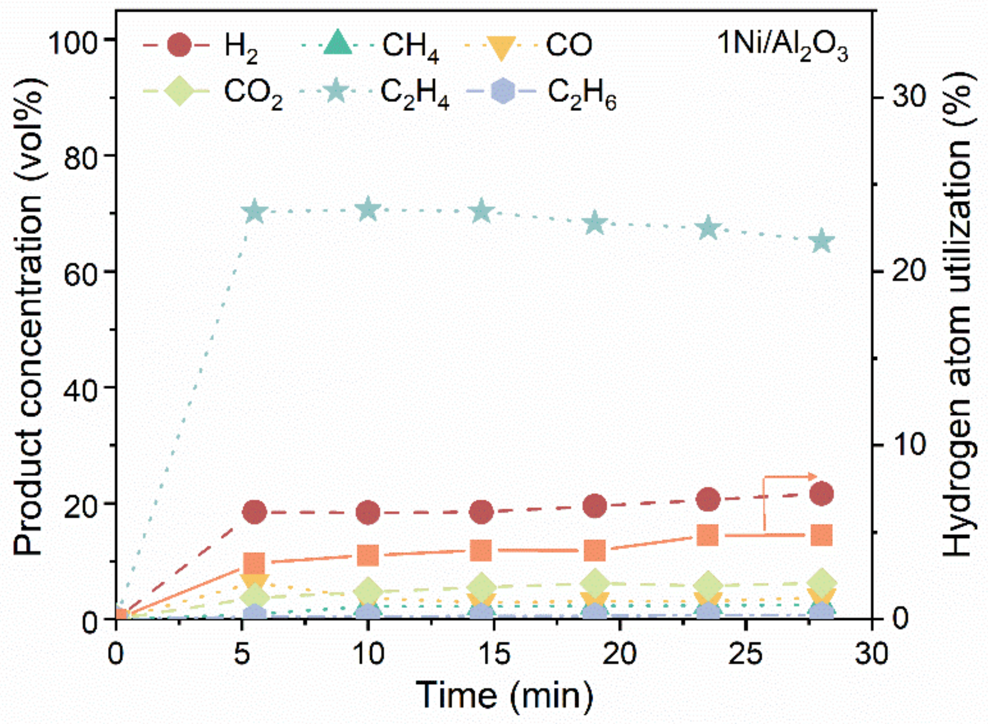

Supplementary Figure 36. SRE performance of 1Ni/Al$_2$O$_3$. Activity test conditions: 1 atm, 550 °C, S/E=3, liquid feeding rate 0.005 mL min^{-1}, N_2 flow rate 40 mL min^{-1}.

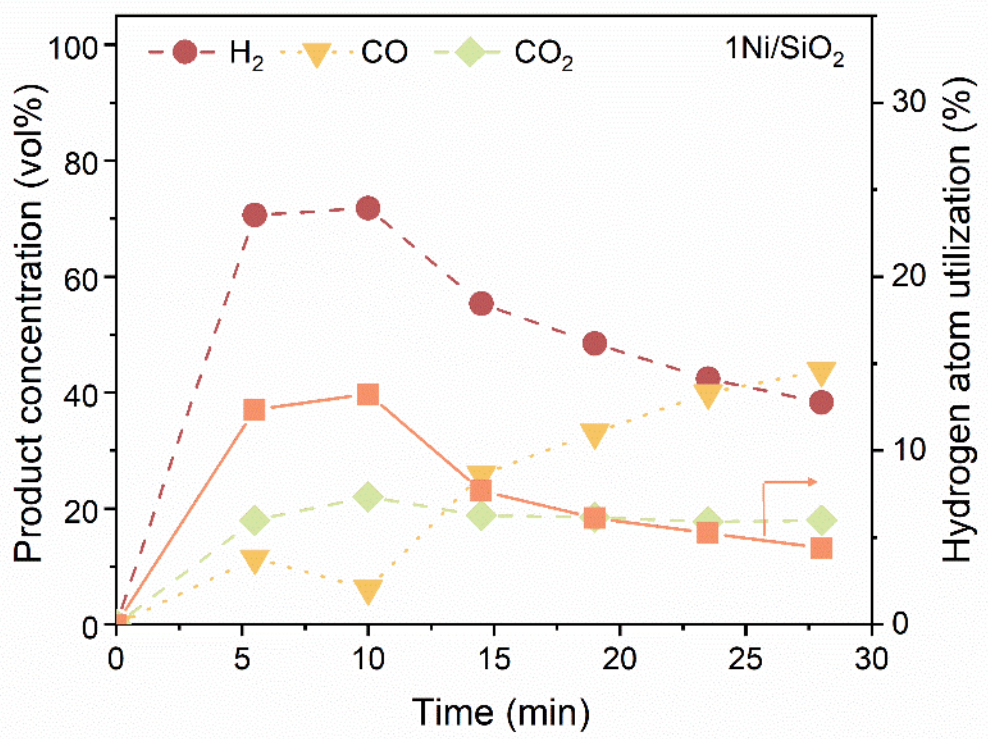

Supplementary Figure 37. SRE performance of 1Ni/SiO_2. Activity test conditions: 1 atm, 550 °C, S/E=3, liquid feeding rate 0.005 mL min^{-1}, N_2 flow rate 40 mL min^{-1}.

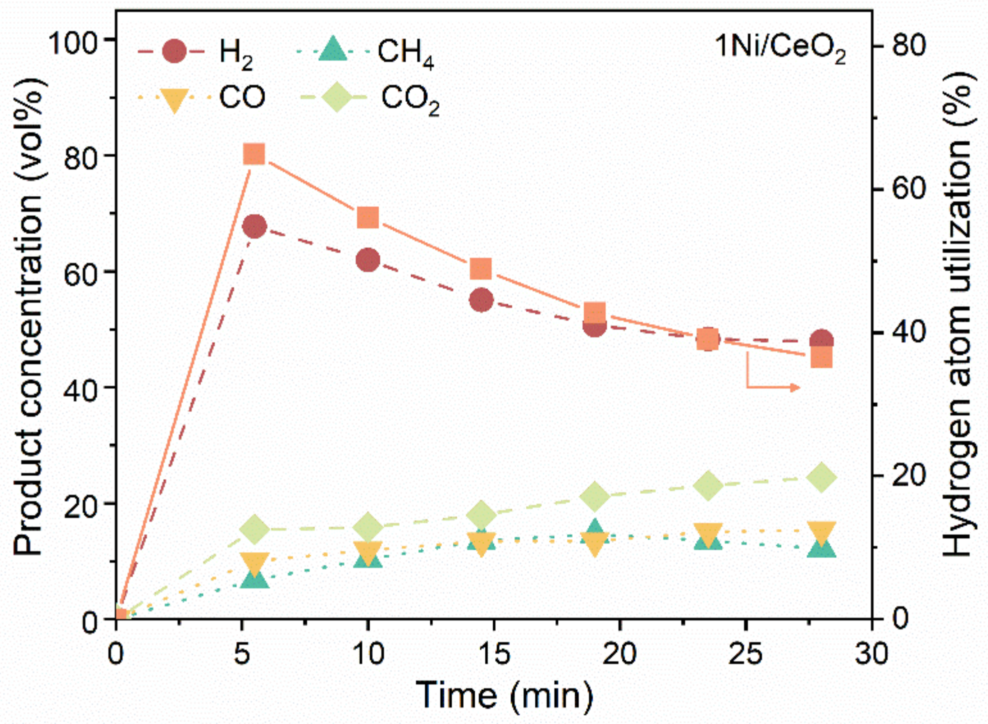

Supplementary Figure 38. SRE performance of 1Ni/CeO_2. Activity test conditions: 1 atm, 550 °C, S/E=3, liquid feeding rate 0.005 mL min^{-1}, N_2 flow rate 40 mL min^{-1}.

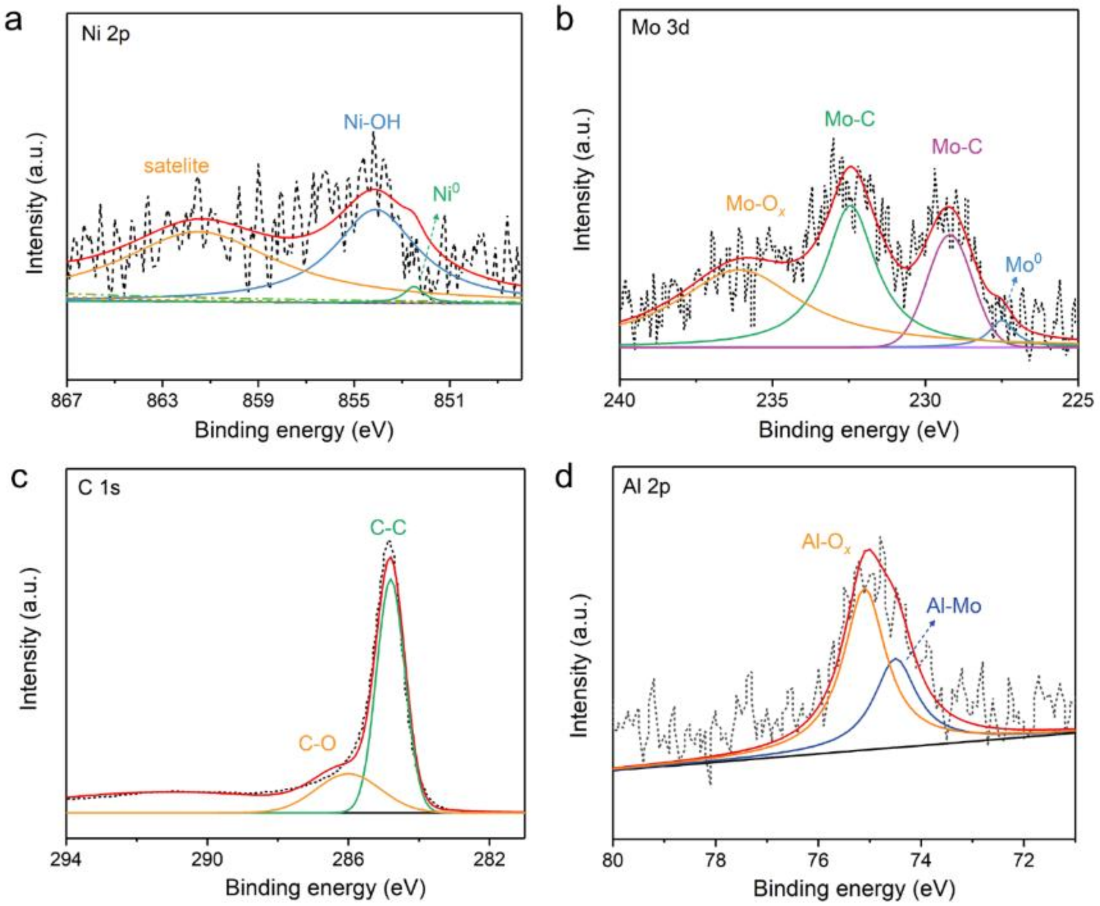

Supplementary Figure 39. XPS spectra of the 120 h reacted 1Ni/MTAC catalyst. a Ni 2*p* spectrum. **b** Mo 3*d* spectrum. **c** C 1*s* spectrum. **d** Ti 2*p* spectrum. **e** Al 2*p* spectrum. **f** O 1*s* spectrum.

The layered structure of MTAC is Mo-C-Ti-C-Mo-Al-Mo-C-Ti-C-Mo; compared to the as-prepared 1Ni/MTAC, the intensity of Al-Mo peak decreased and the characteristic peak of Mo-C disappeared, indicating the formation of Ni-Mo_n-$Mo_{2-n}TiAlC_2$ structure.

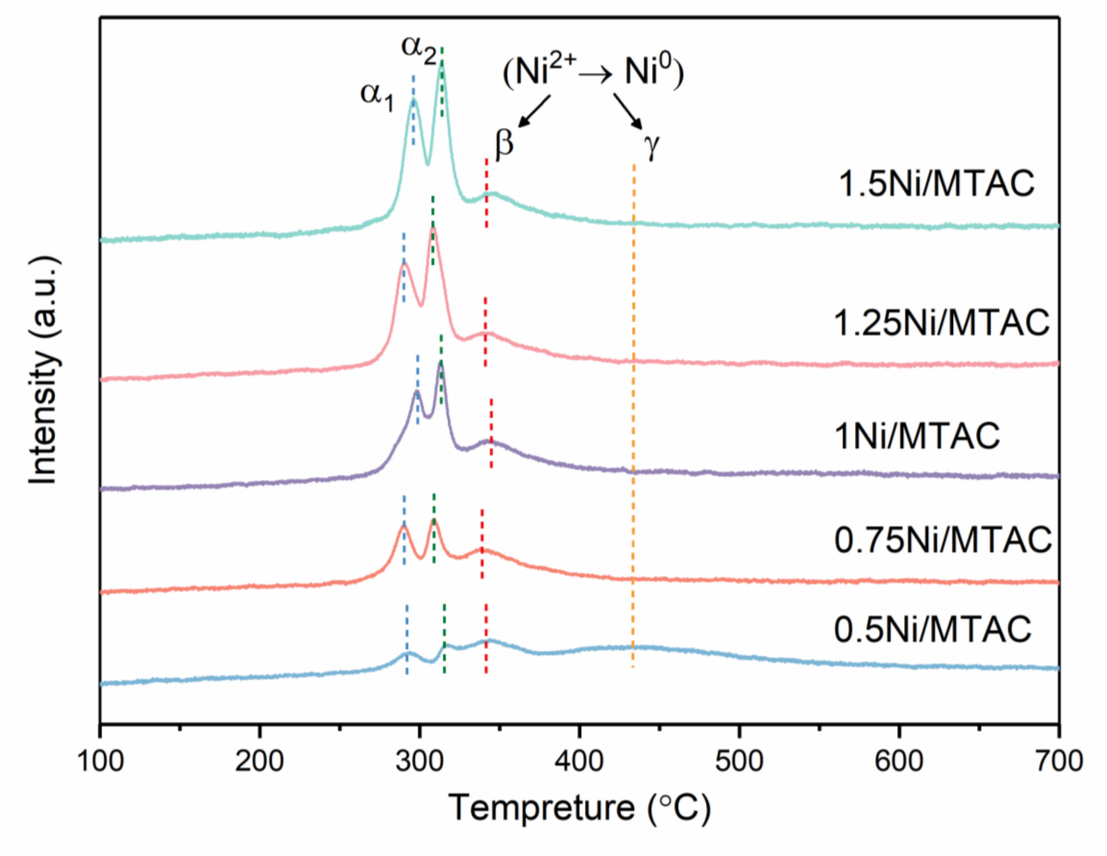

Supplementary Figure 40. H_2-TPR profiles of unreduced Ni/MTAC catalysts.

For the catalysts with different Ni loadings, four hydrogen consumption peaks (α_1, α_2, β and γ) were observed. α_1 and α_2 peaks can be ascribed to the reduction of adsorbed oxygen which is easily reduced by H_2 at low temperatures[5]. β and γ peaks are related to the reduction of nickel oxides, β peak is generally assigned to the reduction of relatively free nickel oxide that weakly interacts with the support surface. Whereas, γ peak is attributed to nickel oxides that strongly interacts with support[6]. In contrast, the reduction temperature of 1Ni/MTAC is slightly higher than those of other catalysts, possibly attributing to the strongest Ni-MTAC support interaction of the Ni doped samples.

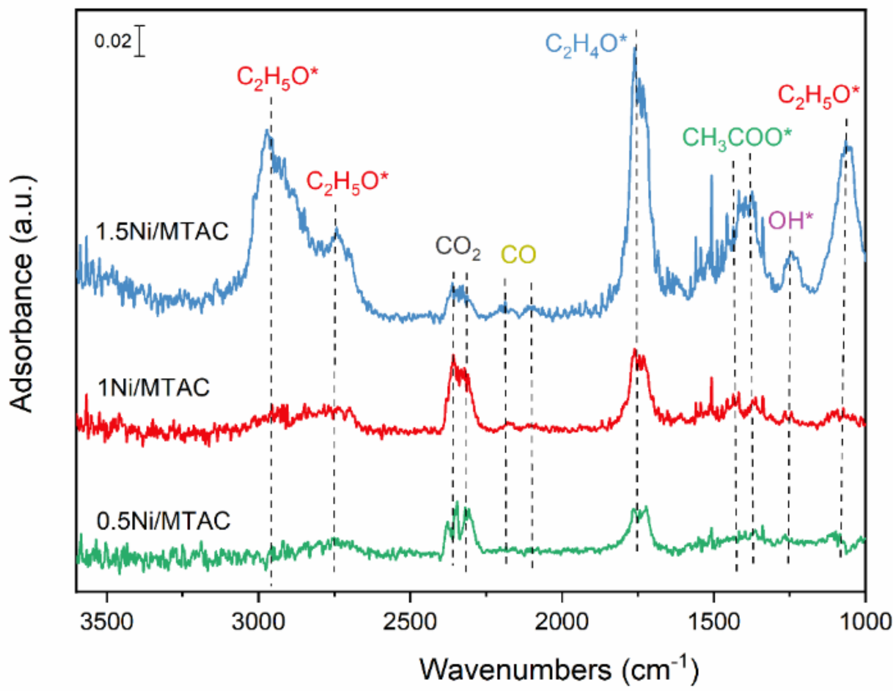

Supplementary Figure 41. Comparisons of TPER experimental results at 350 ℃.

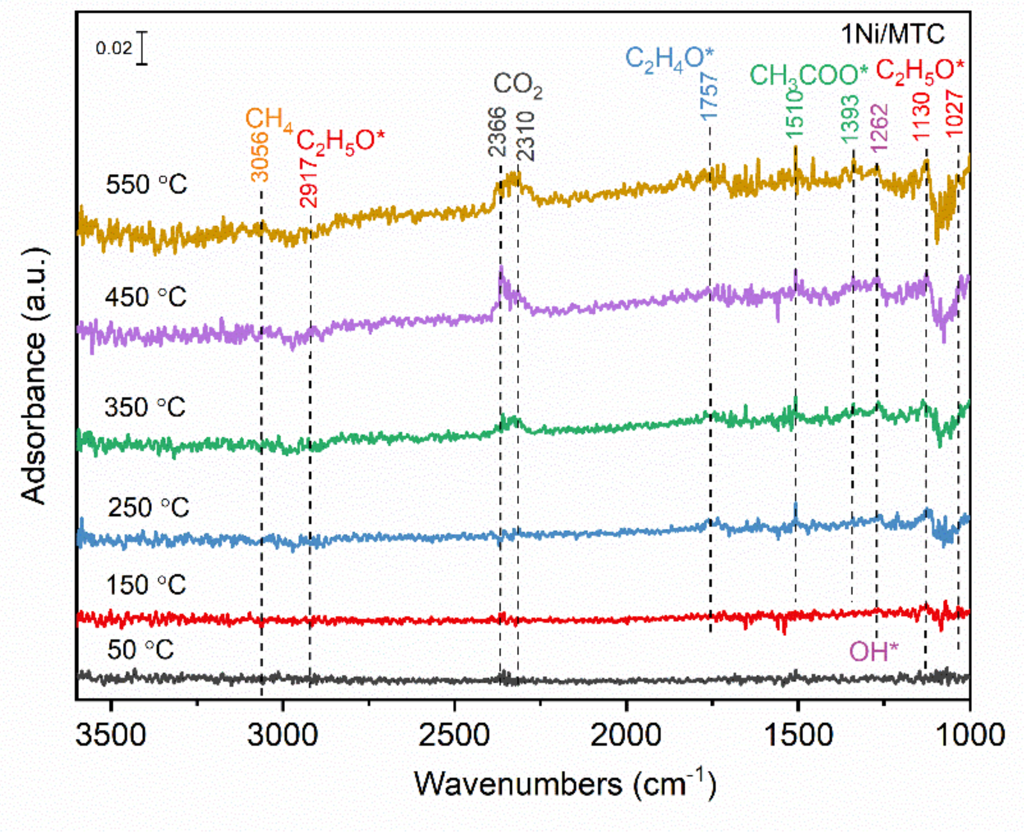

Supplementary Figure 42. In situ DRIFTS of 1Ni/MTC.

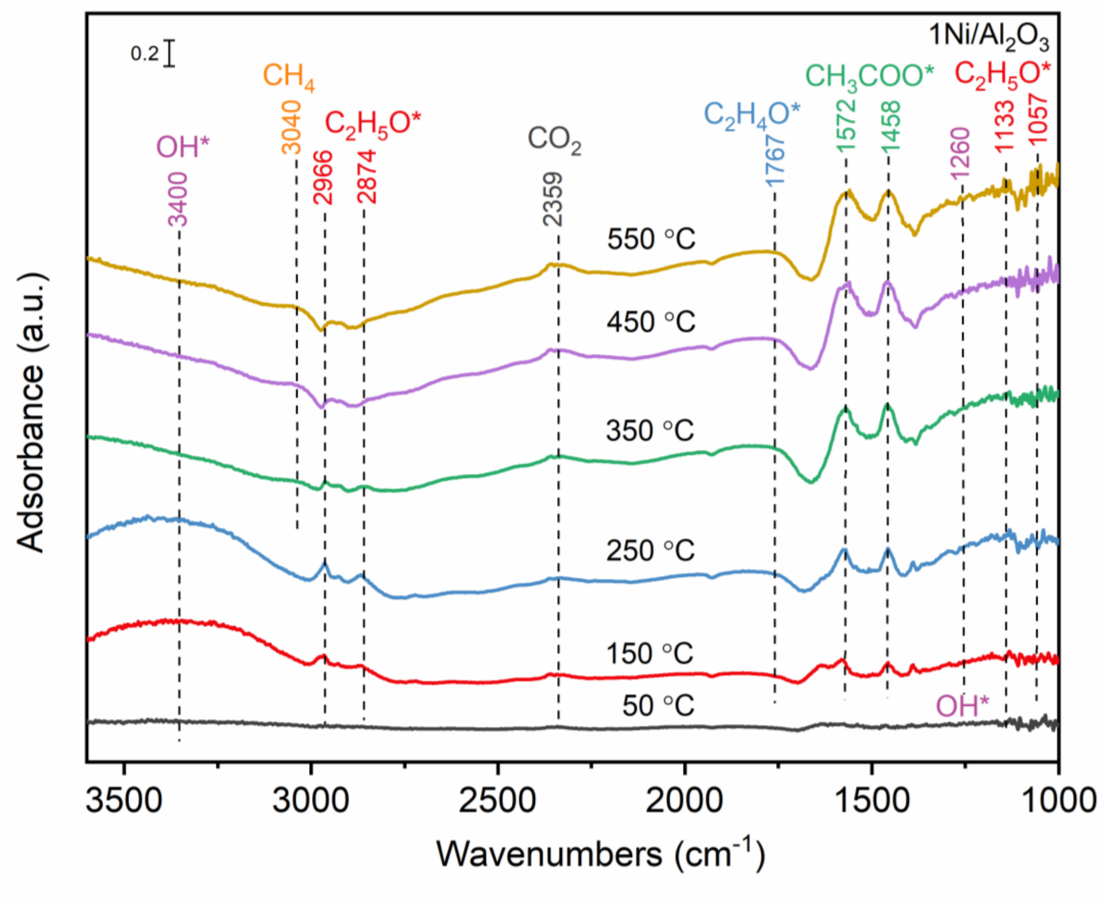

Supplementary Figure 43. In situ DRIFTS of 1Ni/Al$_2$O$_3$.

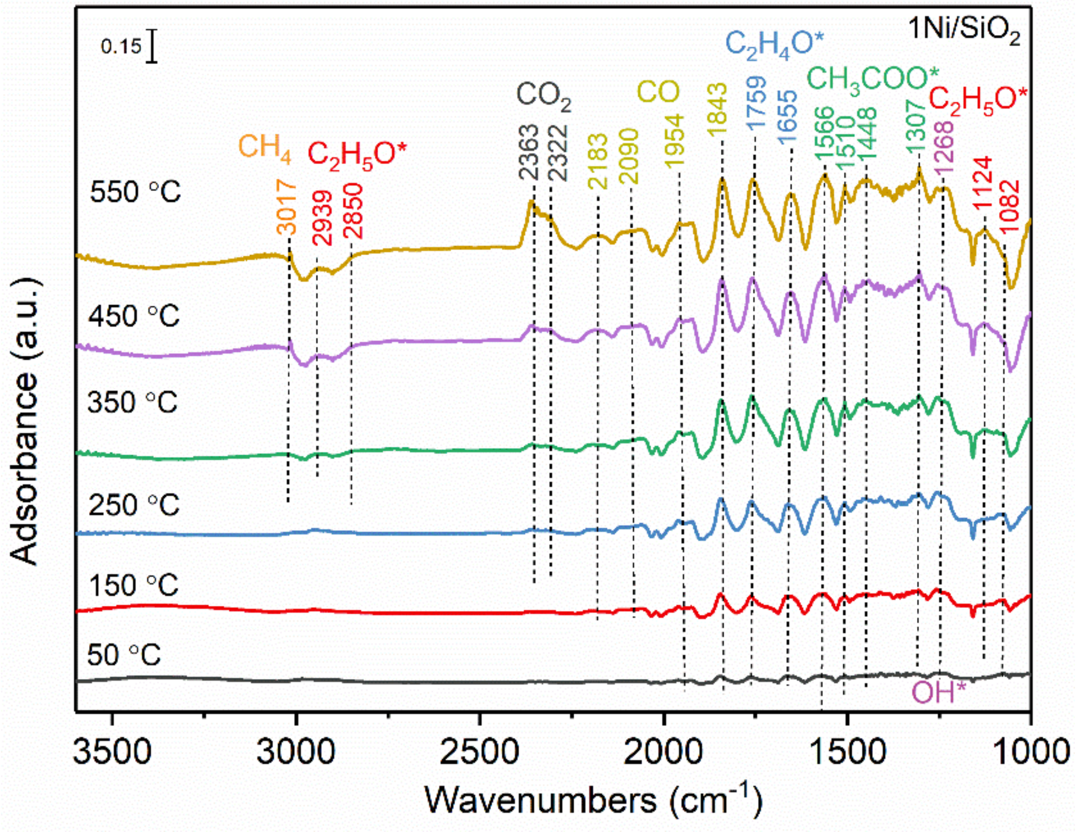

Supplementary Figure 44. In situ DRIFTS of 1Ni/SiO_2.

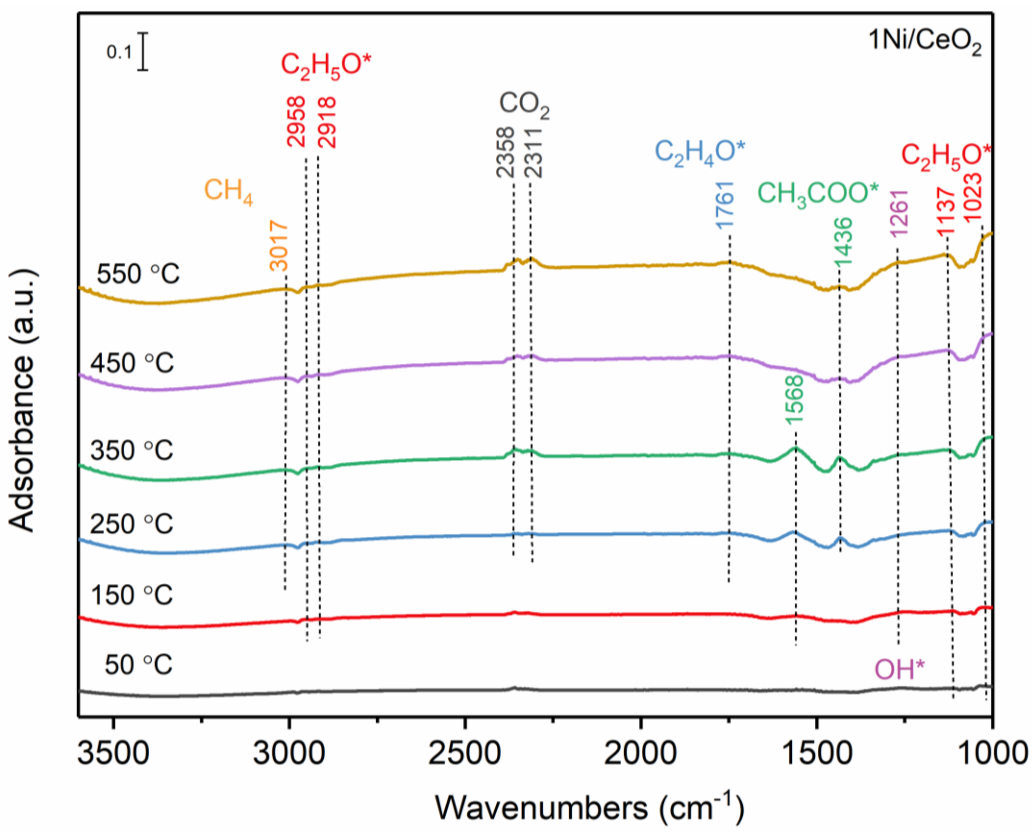

Supplementary Figure 45. In situ DRIFTS of 1Ni/CeO_2.

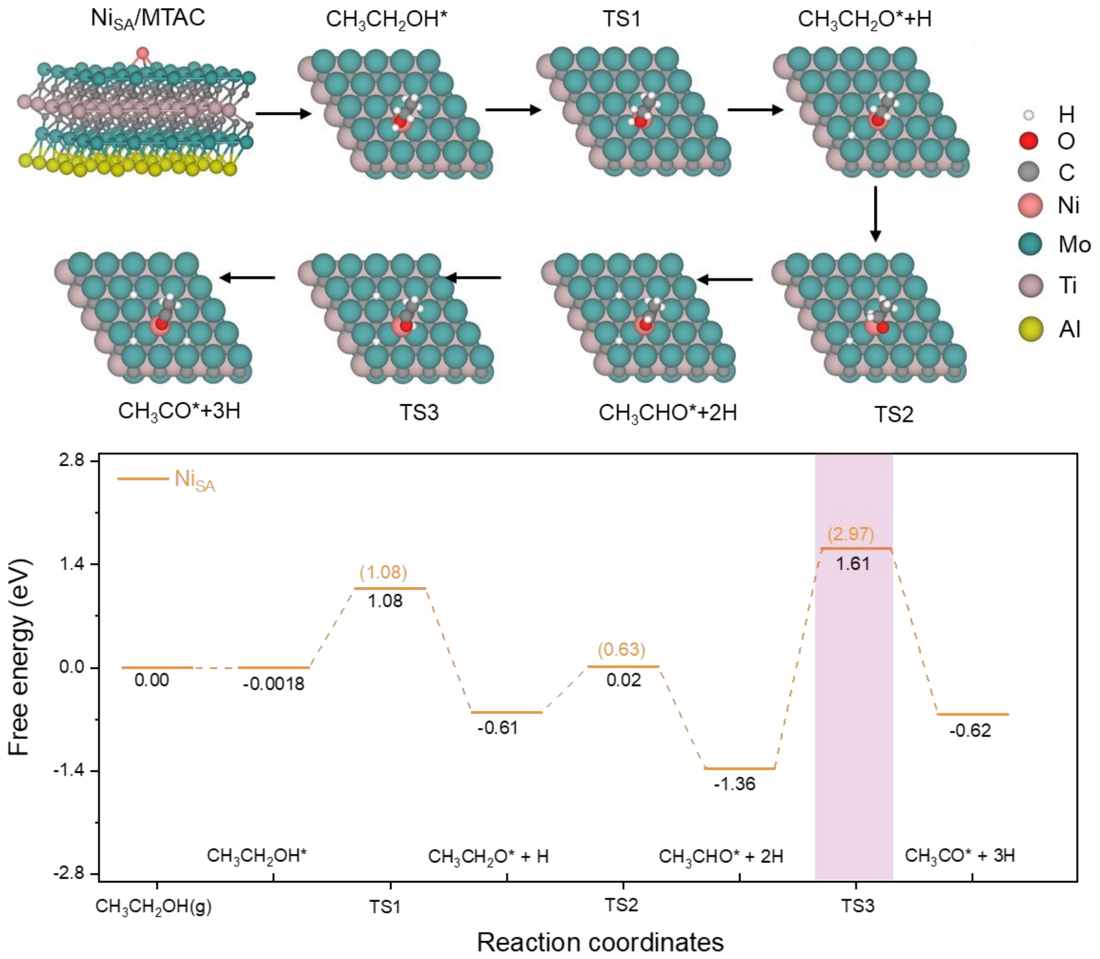

Supplementary Figure 46. DFT studies for CH_3CH_2OH dehydrogenation on Ni$_{SA}$/MTAC(004). Calculated potential energy diagram and corresponding geometric structures for successive dehydrogenation of ethanol molecule on Ni$_{SA}$/MTAC(004). TS represents the transition state. The black and orange numbers denote adsorption energy and reaction energy barrier, respectively.

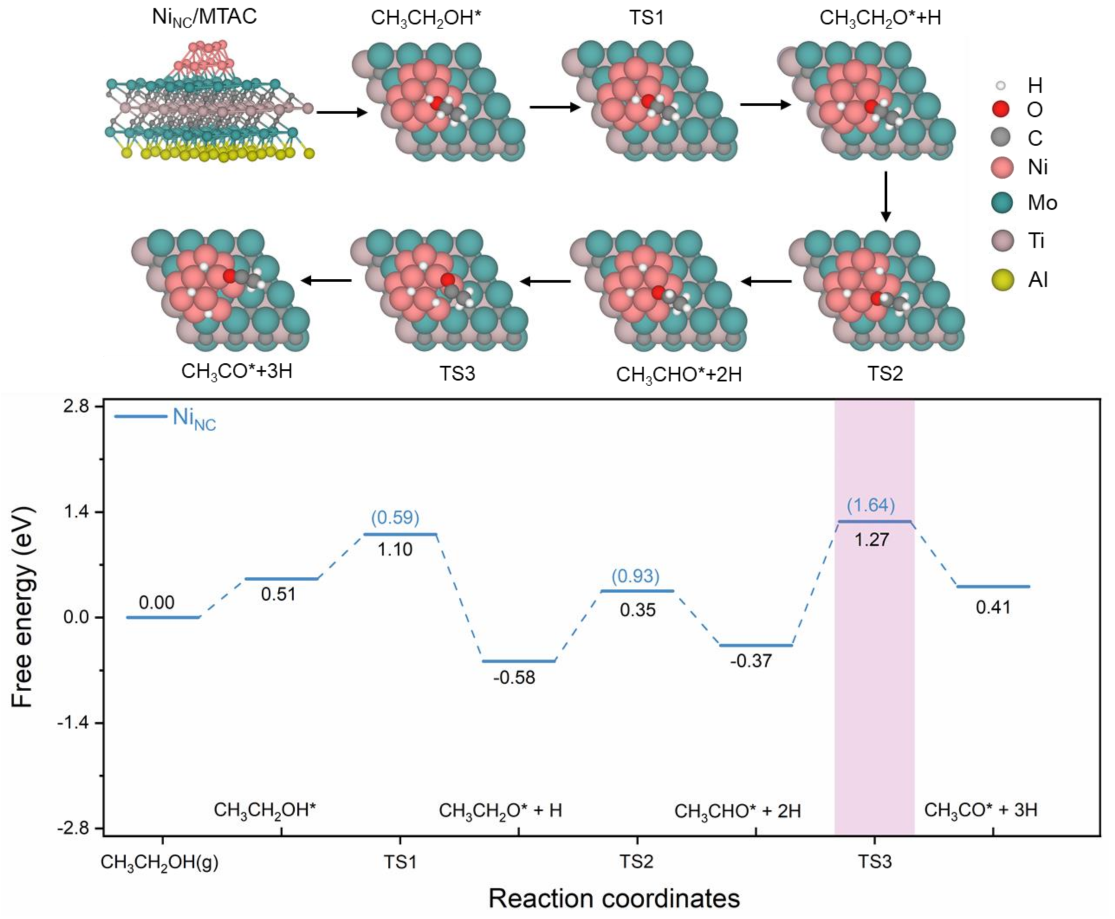

Supplementary Figure 47. DFT studies for CH_3CH_2OH dehydrogenation on Ni_{NC}/MTAC(004). Calculated potential energy diagram and corresponding geometric structures for successive dehydrogenation of ethanol molecule on Ni_{NC}/MTAC(004). TS represents the transition state. The black and blue numbers denote adsorption energy and reaction energy barrier, respectively.

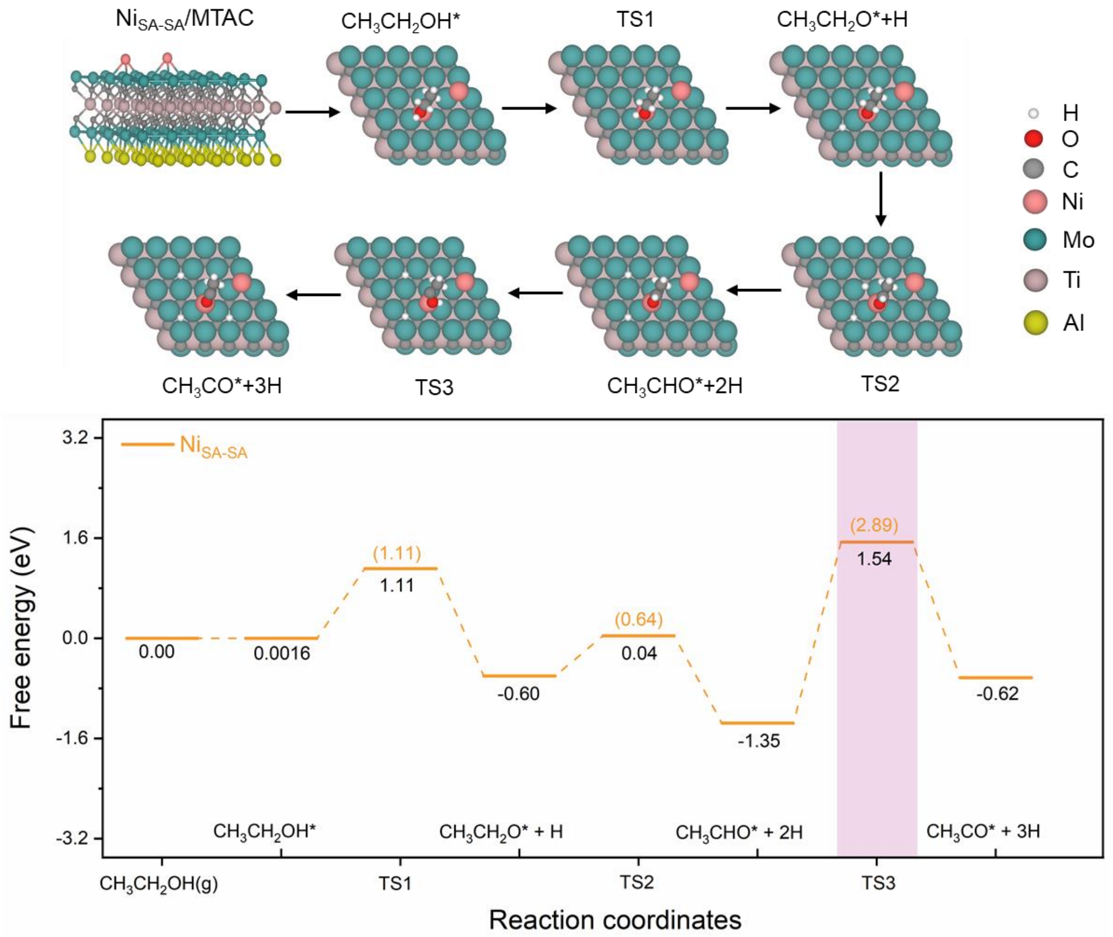

Supplementary Figure 48. DFT studies for CH_3CH_2OH dehydrogenation on Ni$_{SA-SA}$/MTAC(004). Calculated potential energy diagram and corresponding geometric structures for successive dehydrogenation of ethanol molecule on Ni$_{SA-SA}$/MTAC(004). TS represents the transition state. The black and blue numbers denote adsorption energy and reaction energy barrier, respectively.

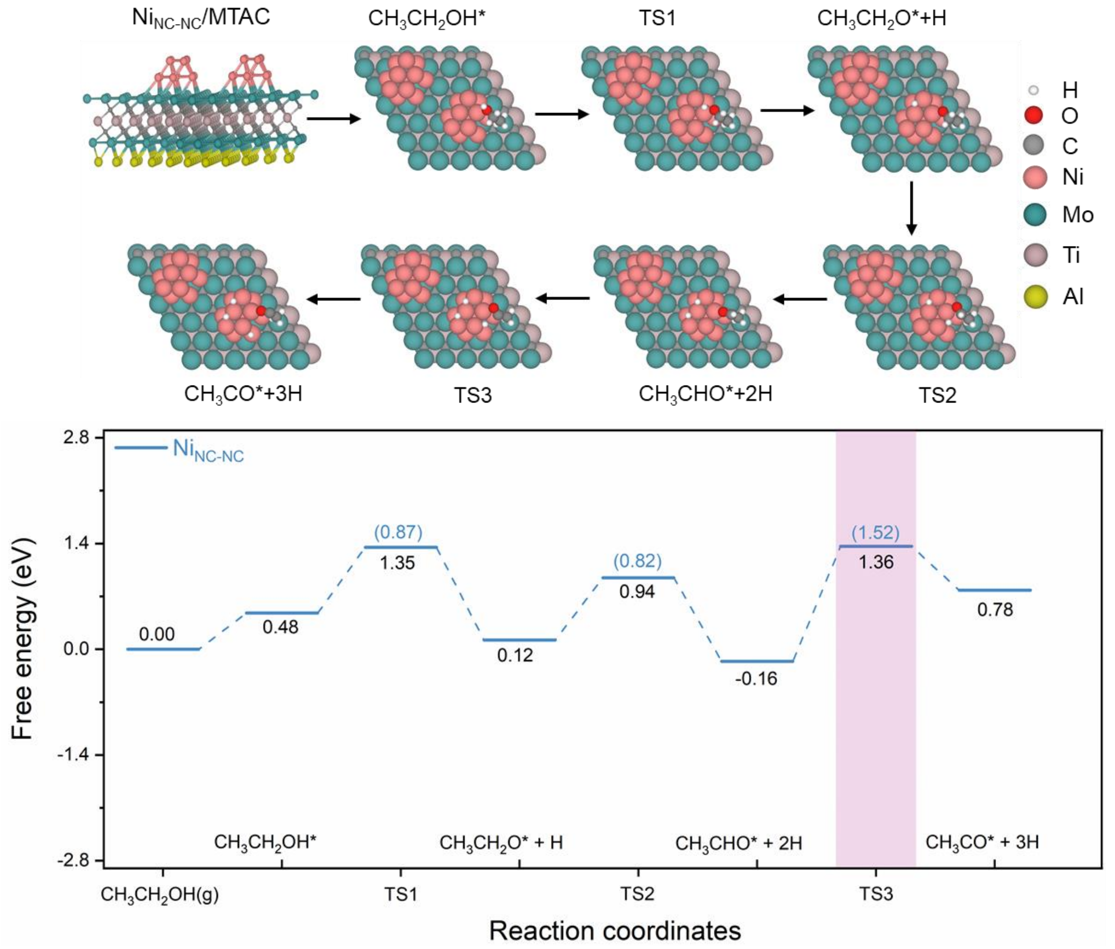

Supplementary Figure 49. DFT studies for CH_3CH_2OH dehydrogenation on Ni_{NC-NC}/MTAC(004). Calculated potential energy diagram and corresponding geometric structures for successive dehydrogenation of ethanol molecule on Ni_{NC-NC}/MTAC(004). TS represents the transition state. The black and blue numbers denote adsorption energy and reaction energy barrier, respectively.

When two Ni single atoms (Ni_{SA-SA}) and two Ni nanoclusters (Ni_{NC-NC}) are introduced, the energy barriers for the transition states remain basically similar tendencies, i.e., the computational results align with our previous findings. This indicates that the adsorption of the intermediate on the supported Ni single atom is still strong, while the introduction of nanocluster modulates an appropriate adsorption for ethanol activation as well as lower the energy barriers for the dehydrogenation of ethanol and other intermediates. Specifically, the barrier of 1.5Ni/MTAC for C_2H_5O* hydrogenation to C_2H_4O* is still high, this is in agreement with the in situ DRITFS results. Besides, the rate-determining step of all catalyst remains the dehydrogenation of acetaldehyde, with the transition state barrier of 0.5Ni/MTAC and 1.5Ni/MTAC

decreasing from 2.97 eV→2.89 eV and 1.64 eV→1.54 eV. Overall, Ni_{SA-NC} models with single atom and cluster co-adsorption is the best, and its potential barrier is the smallest of 1.28 eV. The synergy of single atoms and nanoclusters results in a moderate adsorption of the intermediate species and sharply decreased energy barriers for intermediates dehydrogenation on Ni_{SA-NC}/MTAC, thus boosting the catalytic activity of bio-ethanol reforming.

Supplementary Table 1. Physiochemical properties of the as-prepared samples.

Samples	BET surface area (m^2/g)[a]	Pore volume (cm^3/g)[a]	Pore diameter (nm)[a]
0.5Ni/MTAC	2.3	0.007	3.9
0.75Ni/MTAC	3.4	0.005	3.8
1Ni/MTAC	3.7	0.011	3.8
1.25Ni/MTAC	4.1	0.011	3.9
1.5Ni/MTAC	5.2	0.010	3.9
1Ni/MTC	6.4	0.017	3.9
1Ni/Al_2O_3	187.6	0.226	3.9
1Ni/SiO_2	2.5	0.003	2.0
1Ni/CeO_2	1.7	0.003	3.9

[a] Determined by N_2 adsorption-desorption.

Supplementary Table 2. Nominal and actual loading wt% of as-prepared samples.

Samples	Nominal loading wt%	Actual loading wt%
0.5Ni/MTAC	0.5	0.41
0.75Ni/MTAC	0.75	0.68
1Ni/MTAC	1	0.95
1.25Ni/MTAC	1.25	1.18
1.5Ni/MTAC	1.5	1.42
1Ni/MTC	1	1.03
1Ni/Al_2O_3	1	0.95
1Ni/SiO_2	1	0.98
1Ni/CeO_2	1	1.05

[a] Determined by ICP-AES.

Supplementary Table 3. Relative proportion of the Ni species for the as-prepared catalysts.

Samples	$Ni^{\delta+}/Ni^{\delta+}+Ni^{2+}$ (%)[a]	$Ni^{2+}/Ni^{\delta+}+Ni^{2+}$ (%)[a]	$Ni^{\delta+}/Ni^{2+}$
0.5Ni/MTAC	18.5	81.5	0.227
0.75Ni/MTAC	33.9	66.1	0.513
1Ni/MTAC	36.8	63.2	0.582
1.25Ni/MTAC	38.7	61.3	0.631
1.5Ni/MTAC	39.2	60.8	0.645

[a] Determined by sub-peak areas of XPS spectra.

Supplementary Table 4. Ni K-edge EXAFS fitted parameters for as-prepared catalysts.

Samples	shell	CN[a]	R(Å)[b]	$\sigma^2 \times 10^2$ (Å^2)[c]	ΔE_0 (eV)[d]	R factor[e]
Ni foil	Ni-Ni	12.0*	2.482	0.61	7.6	0.0008
NiO	Ni-O	5.9	2.078	0.51	-3.3	0.0055
	Ni-Ni	12.2	2.949	0.59		
0.5Ni/MTAC	Ni-Ni	6.7	2.477	0.82	2.3	0.0051
	Ni-Mo	2.3	2.720			
1Ni/MTAC	Ni-Ni	8.5	2.489	0.72	6.0	0.0090
	Ni-Mo	1.3	2.790			
1.5Ni/MTAC	Ni-Ni	9.6	2.490	0.81	4.1	0.0052
	Ni-Mo	0.4	2.790			

[a] Coordination numbers.

[b] Bond distance.

[c] Debye-Waller disorder factor.

[d] adsorption edge offset.

[e] goodness of fit.

Supplementary Table 5. Mo K-edge EXAFS fitted parameters for as-prepared 0.5, 1, and 1.5Ni/MTAC catalysts.

Samples	shell	CN[a]	R(Å)[b]	$\sigma^2 \times 10^2$ (Å^2)[c]	ΔE_0 (eV)[d]	R factor[e]
Mo foil	Mo-Mo	8.0*	2.717	0.38	5.3	0.0013
	Mo-Mo	6.0*	3.130	0.34		
Mo_2C	Mo-C	2.7	2.105	0.40	-7.6	0.0148
	Mo-Mo	6.4	2.982	0.75		
0.5Ni/MTAC	Mo-C	3.0	2.083	0.38	2.7	0.0145
	Mo-Ni	4.4	2.788	0.71	-14.5	
	Mo-Mo	6.5	3.237	0.72		
1Ni/MTAC	Mo-C	3.0	2.085	0.43	0.7	0.0175
	Mo-Ni	4.3	2.788	0.71	-13.4	
	Mo-Mo	6.3	3.237	0.71		
1.5Ni/MTAC	Mo-C	2.9	2.073	0.34	0.2	0.0146
	Mo-Ni	4.0	2.789	0.68	-12.9	
	Mo-Mo	5.8	3.237	0.68		

[a] Coordination numbers.

[b] Bond distance.

[c] Debye-Waller disorder factor

[d] adsorption edge offset.

[e] goodness of fit.

Supplementary Table 6. Catalytic performance for SRE reaction over various Ni based catalysts.

No.	Catalyst[a]	Reaciton Tem (℃)	S/E[b]	GHSV (h^{-1})	Lifetime (h)	Sel (%)[c]	Y (%)[d]	Ref.
1	1Ni/MTAC	550	3	3742	120	65	65	This work
2	10Ni-$CePr_{0.20}$	600	4	44240	50	-	-	8
3	5Ni5Co/SBA-15	500	3	-	-	62	-	10
4	0.5Rh62Ni/TiO_2	400	6	16700	300	77	62	11
5	Cu_1Ni_9/YSZ	450	3	-	20	48	27	12
6	12Ni/20MMT-TiO_2	500	10	13200	5	62	55	13
7	$Ni_{0.95}Mo_{0.05}$/SBA-15	450	3	-	-	22	27	14
8	2Ni2Ru-$MnCr_2$	500	4	-	-	30	70	15
9	$Ni_{0.25}Sn/CeO_2$	400	5	57000	20	27	-	16
10	10Ni4.5Co/WO_x-Al_2O_3	600	4	1045	3	72	-	17
12	10Ni10Fe/$MgAl_2O_4$	400	8	59146	15	62	-	18
13	20Ni/20$Ce_xZr_{1-x}O_2$-Al_2O_3	550	6	10619	-	83	-	19
14	$10Ni_3Pt/CeO_2$	400	3	15000	10	-	22	20

[a] Only the highest performing catalysts from the articles referenced are included.

[b] The molar ratio of steam to ethanol.

[c] The selectivity of H_2 was calculated based on the detected products.

[d] The H_2 yield was calculated based on the detected products.

$$S_{H_2} = \frac{F(H_2)}{\sum F(i)_{out}} \tag{1}$$

$$Y_{H_2} = \frac{F(H_2)}{6 \times F(C_2H_5OH)_{in}} \tag{2}$$

Supplementary References

[1] Sun Z. et al. Enabling low-temperature methanol activation via lattice oxygen induced Cu-O-Cr catalysis. *ACS Catalysis* **13**, 13704-13716 (2023).

[2] Chen C. et al. MoS_2-on-MXene heterostructures as highly reversible anode materials for lithium-ion batteries. *Angew. Chem. Int. Ed.* **57**, 1846-1850 (2018).

[3] Maughan P. A. et al. Pillared Mo_2TiC_2 MXene for high-power and long-life lithium and sodium-ion batteries. *Nanoscale Adv.* **3**, 3145-3158 (2021).

[4] Sheng M. et al. In situ electrosynthesis of MAX-derived electrocatalysts for superior hydrogen evolution reaction. *Small* **18**, 2203471 (2022).

[5] Zhou K. L. et al. Platinum single-atom catalyst coupled with transition metal/metal oxide heterostructure for accelerating alkaline hydrogen evolution reaction. Nat. Commun. 12, 3783 (2021).

[6] Jiao L. et al. Non-bonding interaction of neighboring Fe and Ni single-atom pairs on MOF-derived N-doped carbon for enhanced CO_2 electroreduction. J. Am. Chem. Soc. 143, 19417-19424 (2021).

[7] Zhou S. et al. Vacancy-rich MXene-immobilized Ni single atoms as a high-performance electrocatalyst for the hydrazine oxidation reaction. Adv. Mater. 34, 2204388 (2022).

[8] Xiao Z. et al. Engineering oxygen vacancies and nickel dispersion on CeO_2 by Pr doping for highly stable ethanol steam reforming. *Appl. Catal. B* **258**, 117940 (2019).

[9] Das S. et al. Role of lattice oxygen in methane activation on Ni-phyllosilicate@$Ce_{1-x}Zr_xO_2$ core-shell catalyst for methane dry reforming: Zr doping effect, mechanism, and kinetic study. *Appl. Catal. B* **290**, 119998 (2021).

[10] Rodriguez-Gomez, A. & Caballero, A. Bimetallic. Ni-Co/SBA-15 catalysts for reforming of ethanol: How cobalt modifies the nickel metal phase and product distribution. *Mol. Catal.* **449**, 122-130 (2018).

[11] Meng H. et al. A strong bimetal-support interaction in ethanol steam reforming. *Nat. Commun.* **14**, 3189 (2023).

[12] Chen, F. et al. Ni-Cu bimetallic catalysts on Yttria-stabilized zirconia for hydrogen

production from ethanol steam reforming. *Fuel.* **280**, 118612 (2020).

[13] Mulewa, W., Tahir, M. & Amin, N. A. S. MMT-supported Ni/TiO_2 nanocomposite for low temperature ethanol steam reforming toward hydrogen production. *Chem. Eng. J.* **326**, 956-969 (2017).

[14] Kim, D. et al. Dynamic hydrogen production from ethanol steam-reforming reaction on Ni_xMo_y/SBA-15 catalytic system. *Int. J. Energ. Res.* **39**, 279-292 (2015).

[15] E.A. Smal. et al. Spinel-type $MnxCr_{3-x}O_4$-based catalysts for ethanol steam reforming. *Appl. Catal. B* **283**, 119656 (2021).

[16] Tian, H. et al. Role of Sn in Ni-Sn/CeO_2 catalysts for ethanol steam reforming. *Chinese. J. Chem.* **35**, 651-658 (2017).

[17] Contreras JL. et al. Production of hydrogen by ethanol steam reforming using Ni-Co-ex-hydrotalcite catalysts stabilized with tungsten oxides. *Int. J. Hydrogen. Energ.* **46**, 6474-6493 (2019).

[18] Wu Y. et al. Role of Fe species of Ni-based catalysts for efficient low-temperature ethanol steam reforming. *JACS Au* **1**, 1459-1470 (2021).

[19] Wang M. et al. Effect of of active sites distributions on temperature dependent coke formation over Ni/$Ce_xZr_{1-x}O_2$-Al_2O_3 catalysts for ethanol steam reforming: coke precursor gasification. *Appl. Sur. Sci.* **644**, 159746 (2024).

[20] Palma, V. et al. CeO_2-supported Pt/Ni catalyst for the renewable and clean H_2 production via ethanol steam reforming. *Appl. Catal. B*. **145,** 73 84 (2014).
